# A Longitudinal Imaging and Clinical Data Workflow Identifies Potential Time-Dependent Risk Factors for Post-Subarachnoid Hemorrhage Epilepsy

**DOI:** 10.1007/s12028-026-02482-7

**Published:** 2026-03-20

**Authors:** Mitchell Butler, Yuncheng Hao, Jing Wang, Biswajit Maharathi, Anna Serafini, Jensen Wong, Jared Davis, Parin Shah, Fabien Dachet, Joseph R. Geraghty, Fernando D. Testai, Dilip Pandey, Jeffrey A. Loeb

**Affiliations:** 1 Department of Neurology and Rehabilitation, University of Illinois at Chicago, Chicago, IL USA; 2 Department of Mathematics, Statistics, and Computer Science, University of Illinois at Chicago, Chicago, IL USA; 3 Department of Neurology, University of Pennsylvania Perelman School of Medicine, Philadelphia, PA USA; 4 Department of Biomedical Engineering, University of Illinois at Chicago, Chicago, IL USA

**Keywords:** Subarachnoid hemorrhage, Seizures, Epilepsy, Data science

## Abstract

**Objective:**

Subarachnoid hemorrhage (SAH) can lead to recurrent seizures, but the clinical factors that influence which patients will develop epilepsy remain unclear. We built a workflow integrating longitudinal clinical, laboratory, and brain imaging data to identify factors associated with post-SAH epilepsy.

**Methods:**

Retrospective review of electronic medical records followed by direct patient contact was used to identify patients with nontraumatic SAH and determine whether they later developed epilepsy. A total of 58 longitudinal imaging, clinical, hematologic, coagulation, metabolic, and cerebrospinal fluid (CSF) variables were analyzed from the first 14 days following SAH. Blood location and ventricular size were quantified from serial computed tomography images using an automated pipeline. A two-step statistical approach was used to identify time-dependent variables associated with post-SAH epilepsy. Data-driven logistic regression modeling was implemented using features extracted from individual time series to identify which variables could be the most important for determining post-SAH epilepsy risk.

**Results:**

Out of 134 patients, 15 (11.2%) developed post-SAH epilepsy. Patients who developed epilepsy had persistently lower Glasgow Coma Scores, increased total and pericortical blood volumes up to day 8, as well as elevated peripheral eosinophil counts and systemic immune-inflammatory index in the second week up to day 13 following SAH. Exploratory logistic regression analysis identified systemic immune-inflammatory index, total blood volume, and CSF white blood cells as the most influential variables.

**Interpretation:**

We demonstrate the ability of a novel workflow combining longitudinal imaging and clinical data to identify potential risk factors for post-SAH epilepsy. While limited to a single-center retrospective cohort, we observed that pericortical blood in the first week and increased inflammation in the second week after SAH were associated with development of epilepsy. Our approach could be expanded to include more patients with SAH at multiple sites, inform future clinical trials to prevent post-SAH epilepsy, and explore other neurologic disorders.

**Supplementary Information:**

The online version contains supplementary material available at 10.1007/s12028-026-02482-7.

## Introduction

Subarachnoid hemorrhage (SAH is a devastating neurological injury that can occur spontaneously, most often due to rupture of a cerebral aneurysm, or result from trauma. SAH carries significant morbidity for survivors, including the development of recurrent seizures. Long-term epilepsy has been estimated to occur in 3–26% of patients with spontaneous SAH [1]. More recently, larger-scale studies of aneurysmal SAH have reported the development of post-SAH epilepsy in 8–12% of patients [2, 3]. Early (acute) seizures occurring within 7 days of SAH are thought to be more of a direct consequence of the initial injury [4], whereas late seizures (> 7 days after SAH), including post-SAH epilepsy, have been associated with both pathophysiologic and iatrogenic factors, including: intracerebral hemorrhage (ICH), surgical treatment with aneurysm clipping, external ventricular drain (EVD) placement, and aneurysm location [2, 5, 6]. However, to identify high-risk patients and develop preventative treatments, a more thorough understanding of the factors associated with post-SAH epilepsy is needed.

Both the extent and location of bleeding are related to complications after SAH, such as delayed cerebral ischemia [7, 8]. However, the role of blood location has not been extensively studied in relation to post-SAH epilepsy. Direct parenchymal brain injury extending to the cortex has been associated with the development of seizures and epilepsy in patients with intracerebral hemorrhage (ICH) [9], but has not been thoroughly explored in patients with SAH. Nakashima et al. surveyed the clinical, imaging, and electroencephalogram (EEG) features associated with early and late seizures after SAH and correlated findings on neuroimaging with epileptiform changes [4]. However, this analysis was qualitative, primarily examining the effect of large intracerebral bleeds.

In addition to blood volume and location, peripheral and cerebrospinal fluid (CSF) markers of inflammation have been identified as predictors of both early complications and poor outcomes after SAH [10–14]. Growing literature has found relationships between inflammation and epilepsy [15–18]. Elevated serum and CSF cytokines may play a role in epileptogenesis [15]. Composite markers of systemic inflammation such as the systemic immune–inflammatory index have been shown to increase the risk of epilepsy [19] and are linked to complications after SAH [20]. Most of these studies relied on single measurements or a small set of fixed time points.

Patients with SAH are typically cared for in a neurological intensive care unit (neuro-ICU) where large volumes of serial clinical and laboratory data are routinely collected. Leveraging this expansive data with other modalities including imaging can advance our understanding of disease pathophysiology and help develop outcome prediction models that could impact clinical care [21]. Here, we built a workflow combining the rich longitudinal clinical and imaging data collected from the neuro-ICU to identify time-dependent variables that are important for the development of post-SAH epilepsy.

## Methods

### Patient Selection

Approval from the UIC Institutional Review Board was obtained (#2017–0794). Patient selection was conducted in two phases: (1) Preliminary retrospective electronic health record (EHR) review; (2) Patient survey conducted via telephone or mail to determine epilepsy-related outcomes. In phase 1, we identified potential subjects by extensively searching the EMR for any documented discharge diagnoses of SAH and screening for eligible patients admitted under the following ICD-9 codes: 430, 852.1, 803.2, 801.7, 800.7, 852.0, 800.2, 801.70, and 801.29. All patients > 18 years old admitted to University of Illinois Hospital between 1 January 2010 and 31 December 2019 with SAH were initially included in the screening. From this group, we excluded patients who had a prior diagnosis of epilepsy or died prior to discharge. In phase 2, we attempted to contact selected patients by phone or mail to complete a brief survey that took at most 20 min to assess epilepsy-related outcomes. We excluded participants who were unreachable by phone or mail due to lack of available contact information, did not speak fluent English, or had significant mental impairment. Patients who reported having at least one seizure, regardless of semiology, on their posthospitalization survey were included in the epilepsy group, consistent with ILAE criteria [22]. We did not stratify or exclude patients in the post-SAH epilepsy group based on in-hospital seizure activity—whether clinical or electroencephalographic, if present. Those who did not clearly meet these criteria but were on long-term anti-seizure medications (ASMs) at the time of survey were considered their own group and ultimately excluded from this study. All other patients were designated as “controls.” Each patient record was manually reviewed to determine if SAH was nontraumatic or traumatic. We excluded patients with traumatic SAH, which is considered a distinct entity associated with a different pathophysiology and presentation on imaging as well as better outcomes [23]. Thus, in this study, we defined post-SAH epilepsy as the occurrence of > 1 seizure following hospitalization for nontraumatic SAH, regardless of in-hospital seizure activity.

### Data Extraction and Preprocessing

Approval was obtained to review all patient records from their initial admission for SAH up to the present day. For this study, analysis was restricted to data collected during the first 14 days after SAH. The date of SAH was determined on the basis of manual review of clinical notes and defined as day 0.

All available data, including, lab values, vital signs, medications, as well as text of clinical notes and tabular data entries for each patient were automatically extracted from EHR and stored in our multimodal data integration platform called Intuition [24]. Custom scripts were used to extract information, including Glasgow Coma Scale (GCS) and Hunt and Hess (H/H) assessment scores, from clinical notes and validated manually. Clinical note text was automatically searched and manually reviewed to determine additional clinical characteristics: admission H/H score; presence of ICH and/or subdural hematoma on admission; aneurysmal source of SAH; treatment with microsurgical clipping, endovascular coiling, or both; insertion of EVD. H/H > 3 denoted “high-grade” SAH. The occurrence of complications during hospitalization, including vasospasm, cerebral ischemia, or seizures, was determined by the same method. Of note, “vasospasm” included any vasospasm (e.g. angiographic, sonographic); “cerebral ischemia” was determined on the basis of these specific keywords; “seizures” included any in-hospitalization seizure regardless of symptomatology (i.e. electrographic or clinical). Demographic and clinical characteristics were statistically compared between epilepsy and control patients.

Frequency of data collection varied from many measurements per day, such as for vital signs, to a few instances per week, including computed tomography (CT) images. To limit the time resolution while still capturing day-to-day variation, we down-sampled the dataset for each patient to include at most one measurement per variable per day. Repeated measurements on the same day were averaged together. For patients who had multiple CT scans within the first 24 h, the earliest scan was used for day 0. Lab values for peripheral serum or CSF differential white blood cell (WBC) counts were converted from percentage values to raw counts. For a subset of patients with EVDs inserted for several days, serial CSF measurements, including red blood cells (RBC) and WBCs with differential counts, were extracted and analyzed with other variables. Composite markers of systemic inflammation, including the systemic immune–inflammatory index (SII) and the platelet-to-lymphocyte ratio (PLR), were also analyzed. SII combines platelet count (PLT), absolute neutrophil count (ANC), and absolute lymphocyte count (ALC) and is was calculated as: SII = [(PLT × ANC/ALC)/1000], as previously described [20].

We utilized an automated CT image processing pipeline recently developed by our group [25] to obtain serial hemorrhage and ventricular volume measurements. CT images were first intensity-normalized, skull-stripped, and then coregistered to a standard template coordinate space. Established algorithms were used to obtain automated segmentations for lateral ventricles [25] and hemorrhage [26] volumes, which were subsequently coregistered to template space. Hemorrhage volumes were quantified in four regions of interest defined using a custom label atlas: cisternal spaces, pericortical regions capturing sulcal spaces and grey matter areas, parenchymal areas covering white matter and deeper brain structures, and periventricular regions encompassing the space within and proximal to the ventricles. Total blood volume and total lateral ventricular volume were also quantified for each patient. See Supplementary Materials for detail.

Prior to analysis, we excluded variables measured in less than 50% of patients overall or that had more than more than 62% of data missing in order reduce the risk of detecting outlier-driven effects. These were the most restrictive possible thresholds that allowed CT imaging and CSF variables to be included.

### Identifying Variables Associated with Post-SAH Epilepsy

We screened all variables to determine which showed group-level differences between epilepsy and control patients, then selected a subset of variables more robustly associated with post-SAH epilepsy. Selected variables were used for further exploratory logistic regression modeling analysis. The initial screening used a smoothing Spline ANOVA (SSANOVA) analysis as described previously [27]. Briefly, a smoothed curve was fitted to the data across all patients within each group (control or epilepsy) to produce two effective “group mean” curves, and 95% confidence bands for each. Regions where the two bands did not overlap were identified as time windows where control and patients with epilepsy were different. Next, selection was performed using generalized estimating equation (GEE) analysis. This approach, analogous to ANOVA, assesses whether each variable was associated with patient group (epilepsy vs. control), time point (day after SAH), and the group-by-time interaction. For each variable, two distinct GEE models were run: (1) an “additive model” with only the effects of group (i.e. epilepsy or not) and time as predictors; and (2) an “interaction model” containing predictor terms for the independent effects of group and time as well as a third term to capture the group-by-time interaction. Variables were selected if they were associated with patient group in the additive model, interaction model, or both. Selected variables were ranked on the basis of the patient group effect *P*-values, where a smaller *P*-value was designated as a stronger association with post-SAH epilepsy. Finally, time series curves for each variable showing the group-averaged data as well as data for individual patients with epilepsy were plotted together and manually reviewed. We excluded variables from further analysis if group differences between epilepsy and control patients were observed to be driven only by a small number of patients (*n* < 2). All variables including those that did not pass SSANOVA screening were assessed with GEE modeling to compare the consistency of these methods.

### Logistic Regression Analysis

To explore what factors could be most influential for predicting post-SAH epilepsy, we performed a data-driven logistic regression analysis using combinations of the selected variables. Similar to the approach described in [28], functional principal component analysis (FPCA) was used to derive a set of model predictors capturing salient features of the longitudinal data while substantially reducing dimensionality. Models were generated using combinations of FPC scores from the longitudinal variables together with clinical covariates. Multiple imputation was used to incorporate CSF variables. Covariates included those that were significantly different between epilepsy and control patients (*P* < 0.05) as well aneurysm clipping, previously associated with post-SAH seizures [6]. Top-performing models from the longitudinal dataset were compared with simpler models of covariates determined at the time of admission. See Supplemenry Materials and Statistical Analysis (below) for detail.

### Identifying Correlations Between Longitudinal Variables

Pairwise correlations among all variables (not just those implicated in post-SAH epilepsy) were also examined. Variables were grouped into three time windows: days 0–2, days 3–6, and days 7–13. Days 0–2 capture the early brain injury period [29], which has been increasingly regarded as a critical period for determining post-SAH outcomes [29–32]. Days 3–6 cover the remainder of the first week, important for distinguishing early vs. late seizures [4, 9, 33]. Days 7–13 encompass the second week. For each time window, the median value of each variable for each patient was compared with all other variables using Spearman correlation coefficients.

### Statistical Analysis

All data visualization and statistical analyses, including running the SSANOVA and GEE models, logistic regressions, and correlation statistics were done in R [34]. Continuous variables including age were compared via Mann–Whitney *U* tests, and categorical variables such as H/H scores were compared via Chi-squared or Fisher’s exact tests.

For GEE analysis, all longitudinal measurements were regrouped into 2-day time bins by averaging together measurements for adjacent days, starting at day 0. This increased the density of key variables (i.e. imaging and CSF parameters) that were sparser than others and reduced between-day variance within variables. Unlike SSANOVA, GEE accounts for individual- as well as group-level variation over time and is more sensitive to this day-to-day variance. Regrouping to 2-day bins was more appropriate to ensure that the data captured group-level variation reflective of the average behavior of multiple individual patients, rather than stitching together day-to-day measurements from different patients. In this vein, to prevent influence owing to spurious individual measurements, each variable was analyzed independently so that only patients with serial measurements available for least 3 out of 7 time points were included in GEE modeling.

Logistic regression model performance was evaluated and compared on the basis of the pooled c-statistic, (area under the curve (AUC), and associated confidence interval computed using the “psfmi” package [40] in R. Receiver operating characteristic (ROC) curve analysis was performed to illustrate the difference in performance between models. Higher performing models were interpreted as indicating variables (individually or in combination) that were more influential for post-SAH epilepsy prediction. Correlation coefficients were computed together with *P*-values adjusted for multiple comparisons testing, and results were visualized using the “corrplot” package [36] in R.

## Results

### Cohort Characteristics

**Fig. 1:**
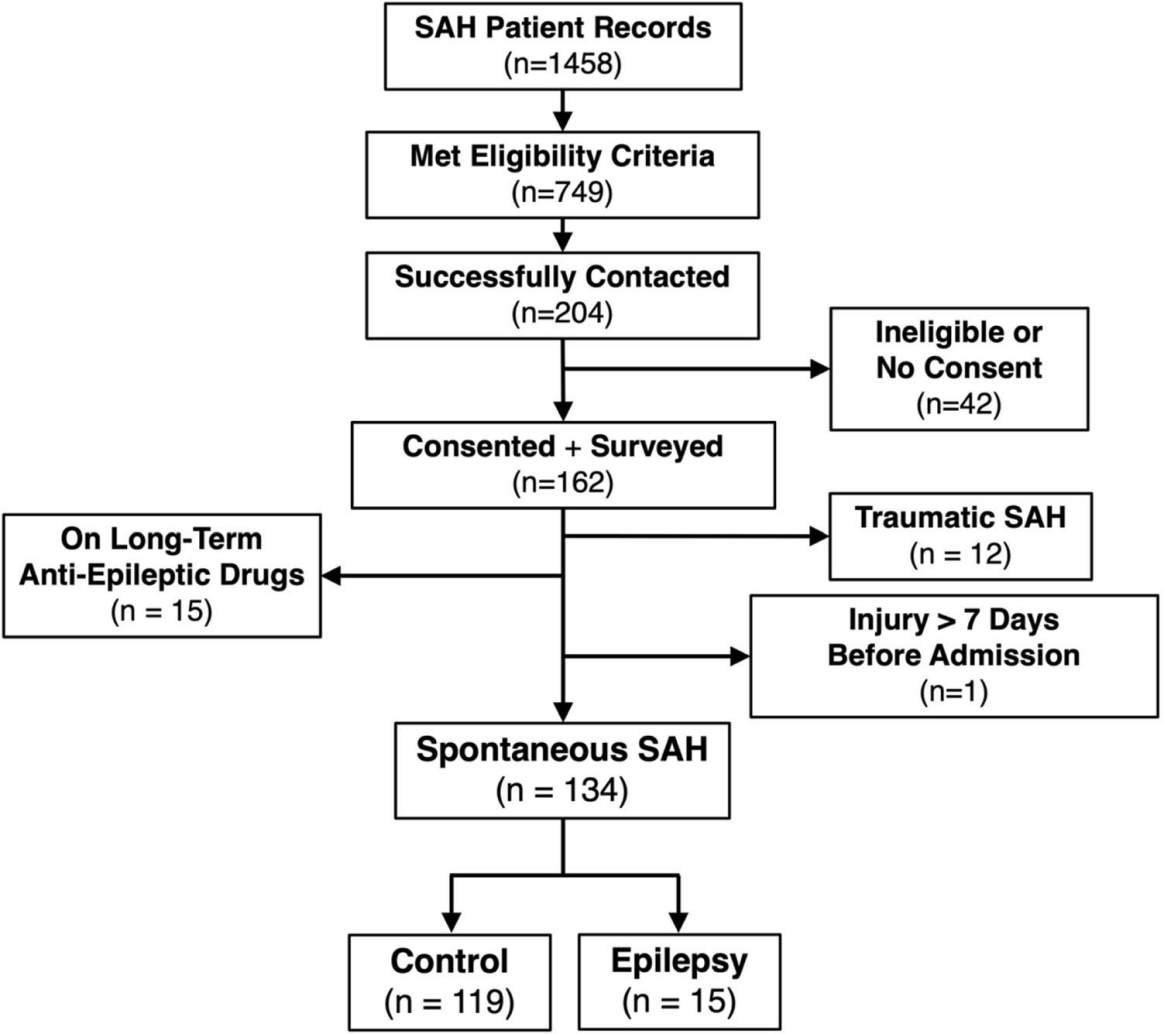
Study design to explore variables associated with post-SAH epilepsy. We screened 1458 records diagnosed with SAH, of whom 749 (51.3%) met initial eligibility criteria. We were able to contact 204 (27.2%) and confirmed eligibility for 162 (79.4%). The final cohort included 134 (82.7%) consented eligible patients with nontraumatic SAH with or without a definitive diagnosis of epilepsy

Figure 1 summarizes our study design and patient inclusion–exclusion criteria. We screened 1458 patients with SAH and identified 749 who met eligibility criteria. Of these, we successfully contacted 204. Among patients we contacted, 42 did not give consent or did not meet eligibility criteria based on information provided on the survey. Consent was obtained for 162 patients to ascertain whether they developed epilepsy and collect information about use of ASMs. All consented and eligible patients completed a structured, standardized questionnaire via telephone interview or mail (see Supplementary Material).

We excluded patients with traumatic SAH (*n* = 12) as well as a second group of patients (*n* = 15) who did not have seizures but reported long-term use of ASMs that could mask the diagnosis. Patients who remained seizure-free but were maintained on ASMs likely include those who are truly seizure-free and those who still would have seizures if they stopped their medications. In our analyses, results for this group were consistently intermediate between the epilepsy and no epilepsy groups, suggesting they could be a mixture of these two more clearly defined patient populations. The final cohort consisted of 134 patients with spontaneous, nontraumatic SAH where 15 (11.2%) developed epilepsy. Supplementary Table S1 shows the SAH etiologies (if determined) for the patients in the epilepsy cohort. Among the small subset (*n* = 3) of patients with confirmed nonaneurysmal SAH, two had ruptured arteriovenous malformations and one patient had no identifiable etiology on MRI or CT angiography imaging.

Table 1 shows that post-SAH epilepsy was associated with presence of ICH on admission (*P* = 0.008) and higher H/H scores (*P* = 0.049) among the subset where data were available. High-grade SAH was more common in patients who developed epilepsy (41.7% vs. 10.9%, *P* = 0.01), in line with previous work [2]. However, we did not observe associations of post-SAH epilepsy with other previously established risk factors, including hypertension (*P* = 0.586), vasospasm (*P* = 0.58), and cerebral ischemia (*P* = 0.20). Further, while the median length of hospitalization was higher for patients with epilepsy (22 days) compared with controls (16 days), this difference did not meet statistical significance (*P* = 0.096).

**Table 1:** Demographic and clinical characteristics of SAH patients who did and did not develop epilepsy

Variable	All (*N* = 134)	No Epilepsy (*N* = 119)	Epilepsy (*N* = 15)	*P* value
*Demographic*				
**Age**	53.5 (45.2–61.8)	54 (44.5–62.0)	49 (46.5–57.0)	0.977
**Female Sex**	84 (62.7)	75 (63.0)	9 (60.0)	> 0.999
**Race**				0.106
Black	61 (45.5)	58 (48.7)	3 (20.0)	
White	38 (28.4)	33 (27.7)	5 (33.3)	
Other	34 (25.4)	27 (22.7)	7 (46.7)	
Unknown	1 (0.7)	1 (0.8)	0 (0.0)	
**Hispanic Ethnicity**	15 (10.4)	12 (10.1)	2 (13.3)	0.657
*Clinical*				
**Past Medical History**				
Diabetes Mellitus	16 (11.9)	15 (12.6)	1 (6.7)	> 0.999
Hypertension	85 (63.4)	74 (62.2)	11 (73.3)	0.571
**Injury at Admission**				
Intracerebral Hemorrhage	19 (14.2)	13 (10.9)	6 (40)	**0.008**
Subdural Hemorrhage^1^	8 (6.0)	1 (6.7)	7 (5.9)	> 0.999
**Hunt & Hess Score**				**0.049**
1	12 (9.0)	11 (9.2)	1 (6.7)	
2	49 (36.6)	45 (37.8)	4 (26.7)	
3	36 (26.9)	34 (28.6)	2 (13.3)	
4	6 (4.5)	5 (4.2)	1 (6.7)	
5	10 (7.5)	6 (5.0)	4 (26.7)	
Unknown	21 (15.7)	18 (15.1)	3 (20)	
**SAH Grade**^**2**^				**0.01**
Low (HH < 3)	97 (85.8)	7 (58.3)	90 (89.1)	
High (HH > 3)	16 (14.2)	5 (41.7)	11 (10.9)	
**SAH Source**				0.552
Aneurysm	94 (70.1)	82 (68.9)	12 (80.0)	
Other	40 (29.9)	37 (31.1)	3 (20.0)	
**Surgical Treatment**^**3**^				
Microsurgical Clipping	64 (47.8)	53 (44.5)	11 (73.3)	0.053^†^
Endovascular Coiling	43 (32.1)	38 (31.9)	5 (33.3)	> 0.999
External Ventricular Drain	70 (52.2)	64 (53.8)	6 (40)	0.413
**In-Hospital Complications**				
Vasospasm	77 (57.5)	67 (56.3)	10 (66.7)	0.582
Ischemia	33 (24.6)	27 (22.7)	6 (40.0)	0.200
Seizures^4^	13 (9.7)	12 (10.0)	1 (6.7)	> 0.999
**Length of Hospitalization**	16.0 (11.0–22.0)	16.0 (11.0–21.5)	22 (13.0–30.5)	0.096

Bolded values dente *P* < 0.05. Continuous variables (Age, Length of Hospitalization) are shown as median (inter-quartile range, IQR). These variables were further explored and ultimately utilized in the exploratory logistic regression analysis

^1^Information not collected for n = 1 patient from the control group

^2^As n = 21 total patients did not have H/H scores available, the group n values are as follows: All (n = 113), No Epilepsy (n = 101), Epilepsy (n = 12)

^3^Note that 91/94 (97%) of SAH patients with aneurysmal source were treated either with clip or coil

^4^Includes seizures at onset, acute (< 7 days after injury, but not at onset) symptomatic (i.e. clinical) and purely electrographic seizures, and late (≥ 7 days after injury) seizures < 7 days. Most patients with in-hospital seizures had seizures at onset of injury, whether No Epilepsy (n = 9, 75%) or Epilepsy (n = 1, 100%)

^†^Treatment with clipping showed a trending difference and was also included in exploratory modeling. See the text

### Identifying Time-Dependent Variables Associated with Post-SAH Epilepsy

**Fig. 2:**
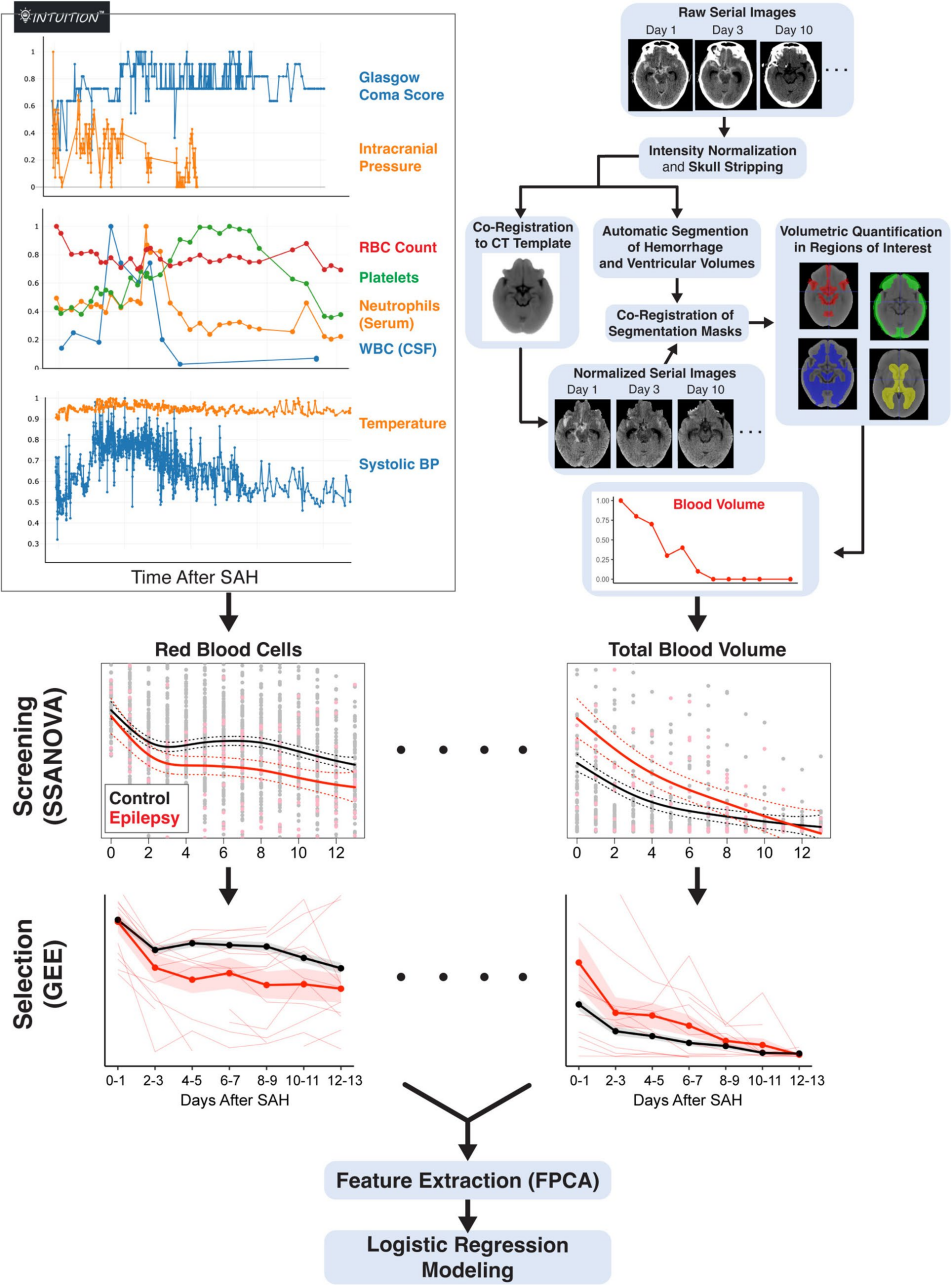
Multimodal data integration and analysis workflow. The top left shows screenshots from our Intuition data platform showing time series curves from a single SAH patient who later developed epilepsy. This includes clinical assessments, laboratory measures, and cerebrospinal fluid measures, and vital signs over 2 weeks. The top right shows an abridged schematic of the image processing and quantification pipeline. On the far right of the schematic are representative images of the CT template with color overlays delineating four anatomical compartments of interest: cisternal [red], pericortical [green], parenchymal [blue], and periventricular [yellow] from which hemorrhage volumes were quantified using a custom label atlas. An example curve of total blood volume vs. time is shown for the same patient whose data are shown on the top left. Example plots show the smoothing spline ANOVA (SSANOVA) analysis time series for blood red cell counts and total blood volume quantified from serial head CT scans. We first screened for significant post-SAH epilepsy variables using SSANOVA to identify potentially significant time-dependent associations of longitudinal variables. Raw data [dots] is plotted together with fitted spline curves [thick solid lines] and associated 95% confidence bands [dotted lines] separately for control [black color] and patients with epilepsy [red color]. Regions where the 95% confidence bands for control and patients with epilepsy are not overlapping are designated as showing a difference between groups. Following SSANOVA, potential predictors of post-SAH were determined using a generalized estimating equation (GEE) modeling using 2-day binned data. Time series for red blood cell count and total blood volume are shown as group means [thick solid lines] ± standard errors [shaded ribbons] together with time series trajectories for individual patients who developed epilepsy [thin red lines]. Functional Principal Component Analysis (FPCA) was then applied to selected variables to extract features from the longitudinal data which were then used as inputs for logistic regression analysis

Serial imaging and multimodal clinical data were analyzed during the first 2 weeks following SAH. Figure 2 summarizes our data workflow that incorporates longitudinal clinical data as well as quantitative brain imaging from serial brain CT scans and illustrates our sequential approach to identify epilepsy-associated variables. Examples are shown on the left for blood red cell counts and on the right for total blood volumes on serial CT scans calculated from our semi-automated imaging pipeline. Both variables passed screening with SSANOVA and show a group-level difference between patients with epilepsy (red line) and controls (black line), but red blood cell count was not selected by GEE analysis.

This two-step screening-then-selection approach was applied to our entire dataset of 58 clinical, laboratory, and imaging variables. Serial CSF measures including red blood cell count, white blood cell count, lymphocytes, and neutrophils were available for a subset of patients (*n* = 64) who had EVDs (of these, *n* = 6 patients developed epilepsy). A total of 27 of 58 variables showed group-level differences between epilepsy and control patients on the basis of SSAVNOVA screening. Of these 27, GEE modeling identified 11 as being strongly associated with post-SAH epilepsy. Supplementary Table S2 lists all 58 variables grouped by data type and shows statistical results for both the SSANOVA and GEE, including the estimated time windows (based on SSANOVA) where epilepsy and control patients are different on a group level. Supplementary Table S3 lists the 11 variables selected by GEE in order of most to least strongly associated with post-SAH epilepsy. Availability of serial data varied substantially across patients and across variables. The number of patients with sufficient data for GEE analysis based on our criteria (see Methods) ranged from 70 total (6 epilepsy + 64 control) patients for CSF variables, up to 147 total (14 epilepsy + 133 control) patients for GCS scores. Serial imaging data was available for most patients (126 total, 14 epilepsy + 112 control). See Supplementary Fig. S1.

#### Quantitative Brain Imaging Variables

**Fig. 3:**
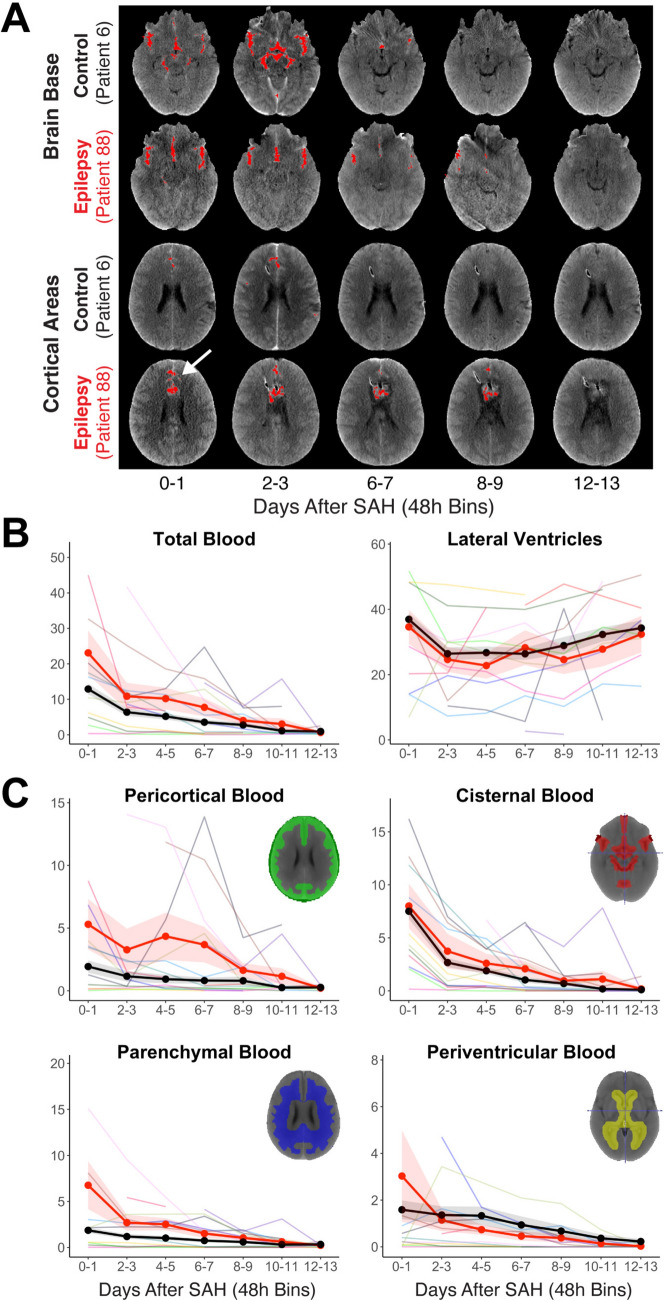
Pericortical SAH blood is most highly associated with post-SAH epilepsy. (**A**) Sample serial normalized images with automatically segmented blood [red] are displayed for both a control and patient with post-SAH epilepsy. Representative slices for the base of the brain showing the cisternal spaces [top half] and higher cortical areas visualizing the sulci and interhemispheric fissure [bottom half] are shown for each patient. In both cases, blood gradually decreases over time, but blood in the higher cortical areas is increased initially [white arrow] and persists longer in the patient who developed epilepsy. (**B**) Time series curves were plotted in 2-day bins for total blood and lateral ventricle volumes. Thick curves with shaded ribbons denote the mean ± standard error for control (*n* = 98) [black] and patients with epilepsy (*n* = 14) [red]. Thin colored curves represent trajectories for individual patients with epilepsy. (**C**) Time series curves for regional blood volumes plotted as in (B) are shown together with inset images of the CT Template with color overlays to depict the pericortical [green, top left], cisternal [red, top right], parenchymal [blue, bottom left], and periventricular [yellow, bottom right]. All *y*-axis values (volumes) shown are in mL, and values are truncated at the 97th percentile

Volumetric data were analyzed from 848 total noncontrast CT images with a mean of 6.38 images per patient obtained over a mean of 11.1 days following SAH. Figure 3A shows normalized CT images from one patient who developed epilepsy and one control patient who did not. Coregistered CT slices are shown together with automated SAH segmentations to demonstrate the evolution of hemorrhage over time near the base of the brain as well as higher cortical areas. In both patients, early blood was present mostly within cisternal spaces; however, the patient who developed epilepsy had more extensive blood in higher cortical areas for up to 9 days after the initial injury. The patients who developed epilepsy had higher total blood volumes particularly in the first few days (Fig. 3B), potentially up to day 8 after SAH (Supplementary Table S2). As shown in Fig. 3C and Supplementary Table S2, cisternal blood volume was not different between epilepsy and control patients, but in those who developed epilepsy, pericortical blood volume was elevated both initially and through the first week up to day 9 following SAH. Parenchymal blood was also elevated in the epilepsy group up to day 5 (Supplementary Table S2), but we observed this primarily in the first 48 h (Supplementary Table S2, Fig. 3C). Lateral ventricular volumes, a measure of cerebral edema, fluctuated, but did not differ between epilepsy and control patients (Fig. 3B).

#### Longitudinal Clinical Variables

**Fig. 4:**
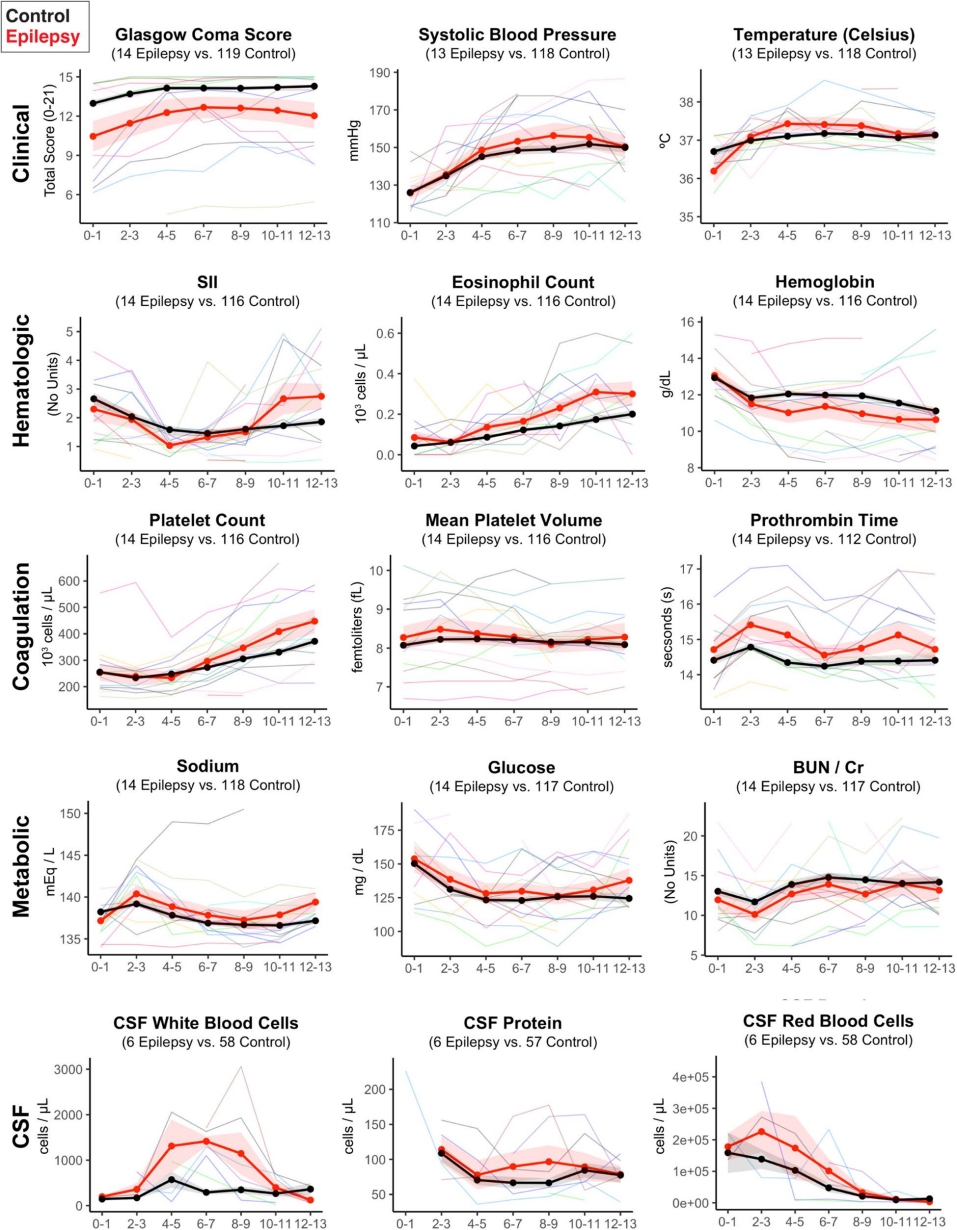
Multiple longitudinal variables are associated with post-SAH epilepsy at different time points. Time series curves plotted in 2-day bins for five categories of longitudinal variables demonstrate clinical, hematologic, coagulation, metabolic, and CSF variables with significant differences in patients with post-SAH epilepsy. Thick curves with shaded ribbons denote the mean ± standard error for control [black] and patients with epilepsy [red]. The number of patients in each group represented is displayed on each plot. Thin colored curves represent trajectories for individual patients with epilepsy. Only patients with at least three repeated measurements were included for each variable. The variables in the leftmost column were found to be the most significant by both SSANOVA and GEE analyses (“selected”). All except sodium were ultimately included in the logistic regression analysis. See the Supplementary Material (Supplementary Fig. S1) for a comprehensive set of similar plots for all remaining variables

We analyzed all the non-imaging longitudinal data consisting of clinical, hematologic, coagulation, metabolic, and CSF variables (Supplementary Table S2). Figure 4 shows samples from each of these categories for control and patients with epilepsy that were strongly associated with post-SAH epilepsy. Total GCS scores were consistently lower in patients with epilepsy. While eosinophil counts were similar between patients with epilepsy and controls early after the SAH, they were increased and remained elevated at later time points. Another measure of systemic inflammation, the SII, also became substantially elevated in patients with epilepsy at later time points.

CSF WBCs had one of the largest group effects, with patients with epilepsy exhibiting substantially elevated levels 4–9 days after SAH. CSF RBCs were elevated in patients with epilepsy early on between 0–3 days after injury. Other variables that did not show clear differences between control and patients with epilepsy included systolic blood pressure, body temperature, mean platelet volume, BUN-to-creatinine ratio (BUN/Cr), glucose, and CSF protein. Several variables that passed screening with SSANOVA, including hemoglobin, prothrombin time, and platelet count (see Fig. 4), were ultimately not selected by GEE analysis.

### Logistic Regression Models Combining Different Types of Variables

**Fig. 5:**
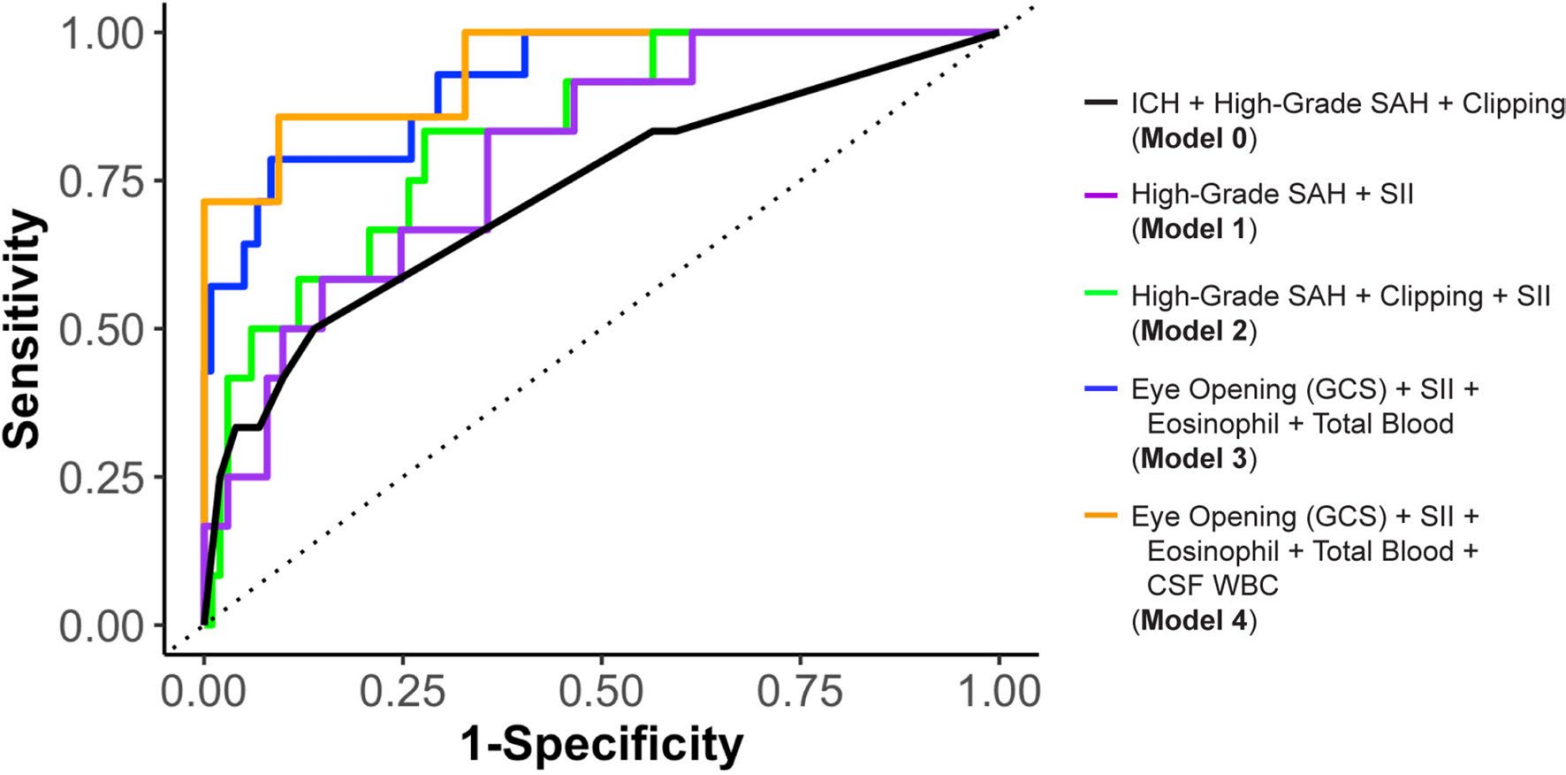
Data-driven logistic regression modeling suggests inflammation and blood volume as key variables that could predict post-SAH epilepsy. ROC curves for predicting post-SAH epilepsy are shown. Models 1 [Purple], 2 [Green], 3 [Blue], and 4 [Orange] comprise a subset of the highest-performing experimental models incorporating longitudinal variables. Model 0 [Black] is a baseline model consisting of clinical indicator variables alone. This was the best-performing indicator-only model and included all three significant clinical covariates: ICH on admission, “high-grade” SAH (H/H > 3), and aneurysm treatment with clipping. Model 3 achieved performance in the 95th percentile across for all models (training AUC > 0.89), and contained the lowest number of predictors (*n* = 8) in that group. Model 4 is similar to Model 3 with the addition of CSF white blood cells (WBC) and showed the highest performance. The average *c*-statistic values (training AUCs) of Models 0–4 in order were: 0.728, 0.782, 0.820, 0.906, and 0.914. The dotted line is the reference (AUC = 0.5)

We performed data-driven logistic regression analysis to identify which variables could have the strongest influence on post-SAH epilepsy prediction. We fit nearly 1800 logistic regression models using unique combinations of 11 selected variables (Supplementary Table S3): eosinophil count, CSF red blood cell count; blood volumes (total and pericortical); GCS total, verbal, and eye-opening scores; composite peripheral inflammatory indices (SII and PLR); and CSF inflammatory markers (total WBCs and neutrophils). After excluding those that did not meet our inclusion criteria, we analyzed 1771 models. Figure 5 shows the ROC curves of the five best-performing models and demonstrates the value of combining multiple types of longitudinal variables.

The simplest model (Model 0) which included only ICH on admission, high-grade SAH (H/H > 3), as well as aneurysm treatment with surgical clipping had an AUC of 0.729. Including longitudinal variables significantly increased AUC. Model 1 that included high-grade SAH and SII showed a mean [95% CI] *c*-statistic (training AUC) of 0.782 [0.631–0.883]. Adding additional variables in Model 2 which consisted of Model 1 + clipping was the best four-predictor model with mean [95% CI] *c*-statistic of 0.820 [0.667–0.912]. Models 3 and 4 were in the 95th percentile across 1771 experimental models with mean [95% CI] *c*-statistics of 0.906 [0.802–0.958] and 0.914 [0.804–0.965], respectively. Incorporating CSF measures further enhanced performance in Model 4 (Fig. 5).

### Exploring Time-Dependent Pairwise Correlations between Variables

**Fig. 6:**
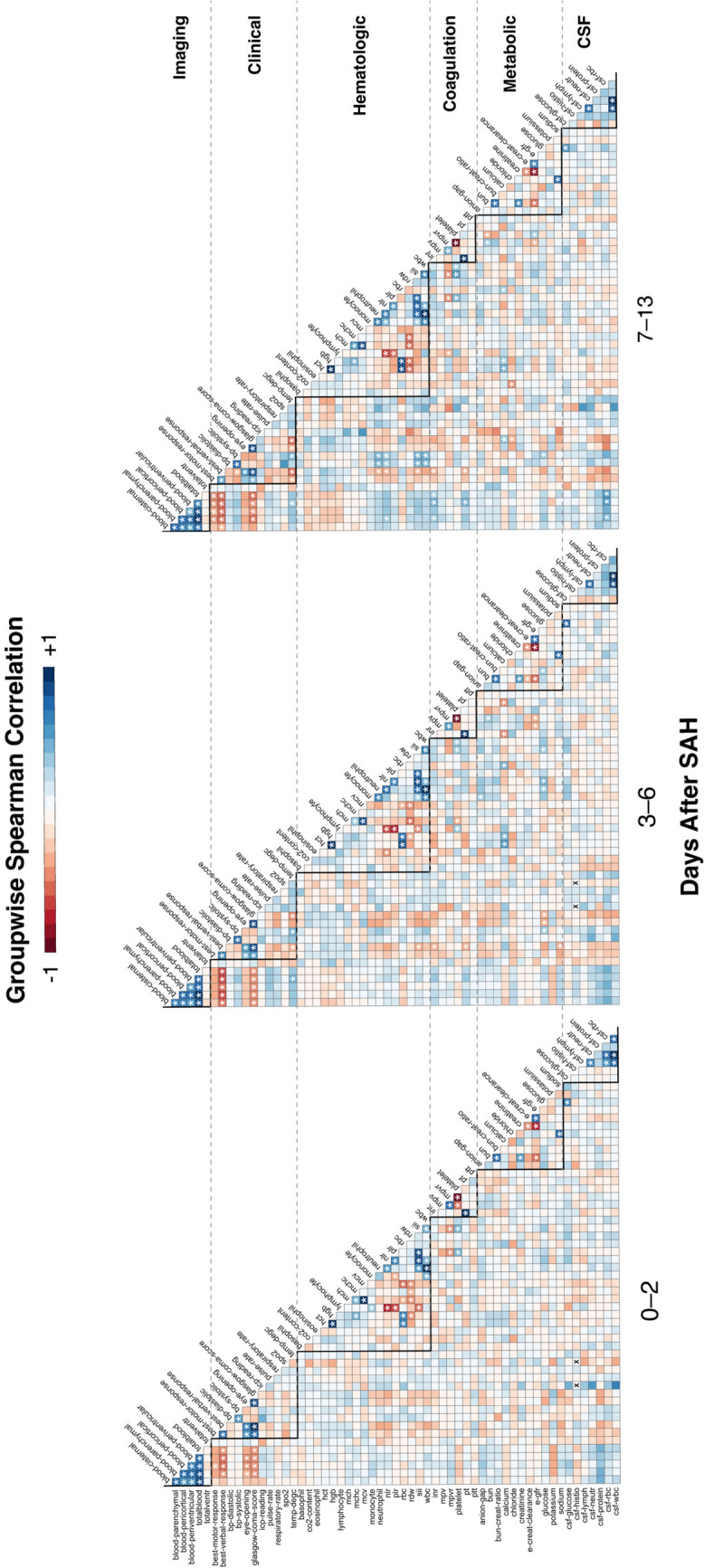
Exploratory correlation analysis identified significant time-dependent relationships between variables. Each colored heatmap shows pairwise Spearman correlations for more than 60 variables during three distinct time windows. Days 0–2 encompass the early brain injury period; days 3–6 capture the rest of the first week; and days 7–13 capture the second week, which includes relevant time windows for development of delayed cerebral ischemia as well as important windows for epilepsy-associated variables. Solid black lines delineate variables within a given category [right] from those outside of it [left]. Darker colors correspond to stronger correlations, with red indicating a negative and blue positive correlations, The asterisks denote statistically significant correlation coefficients of *P* < 0.05, adjusted for multiple comparisons. White boxes with a black X denote missing values for the correlation coefficients due to lack of overlap in available data for a given pair of variables

While the major focus of this study was to identify variables associated with post-SAH epilepsy, we leveraged our workflow to explore pairwise relationships among the entire list of longitudinal variables. We examined group-level pairwise correlations between variables at three discrete time windows as shown in Fig. 6. Complete data for all significant (multiple comparisons-adjusted *P* < 0.05) correlations are shown in Supplementary Table S4. We ultimately observed several statistically significant correlations that were time-dependent. For example, total blood volume was negatively correlated with GCS persistently across days 1–3 (*r* = −0.44, *P* = 0.001), days 4–7 (*r* = −0.48, *P* < 0.001), and days 8–14 (*r* = −0.5, *P* < 0.001). On the other hand, GCS scores were negatively correlated with cisternal blood volume only during days 4–7 (*r* = −0.40, *P* = 0.01), but were persistently, and more strongly, negatively correlated with pericortical blood volume across days 1–3 (*r* = −0.49, *P* < 0.001), days 4–7 (−0.46, *P* < 0.001), and days 8–14. (−0.43, *P* = 0.003). The case was similar for parenchymal and periventricular blood volumes (see Supplementary Table S4). SII was negatively correlated with GCS verbal scores only after the first 72 h on days 4–7 (*r* = −0.36, *P* = 0.03) and days 8–14 (*r* = −0.38, *P* = 0.01), and directly correlated with systolic (*r* = 0.46, *P* < 001) and diastolic (*r* = 0.44, *P* < 001) blood pressures only during the second week (days 8–14) after SAH.

## Discussion

### Inflammation as a Potentially Treatable Risk Factor after Post-SAH Epilepsy

Post-SAH epilepsy was associated with a delayed (> 1 week after injury) increase in systemic inflammation, as indicated by eosinophil counts and SII. In a subset of patients who required EVD placement and had serial CSF analyses, we observed a similarly delayed increase in CSF inflammation, as reflected by elevated WBC counts. These results are consistent with a recent animal study from our group showing that SAH leads to both localized and highly diffuse inflammation throughout the brain associated with marked changes in the electroencephalogram [37]. In this study, there was extensive microglial activation not only localized to the site of hemorrhage, but extensively throughout the cerebral cortex.

Previous work from our group and others has shown that peripheral inflammatory markers, including SII, PLR, neutrophil-to-lymphocyte ratio, and mean platelet volume ratio, can predict which patients with SAH will develop complications and poor outcomes [38–40]. Our results suggest SII as the most influential inflammatory risk factor for post-SAH epilepsy, more so than PLR, neutrophil-to-lymphocyte ratio, or mean platelet volume ratio, which were also explored here. This is interesting given that SII represents, in some sense, the broadest composite inflammatory marker in that it incorporates key components of the others. Further, the association of persistent eosinophilia with post-SAH epilepsy observed here was unexpected. However, post-SAH epilepsy is independently associated with poor functional outcome [41] which has been linked to peripheral eosinophilia [42]. Together, these findings support a growing group of studies suggesting inflammation as a possible therapeutic target to prevent the development of epilepsy [15, 17, 43]. However, it should be noted that the interplay between delayed cerebral ischemia and inflammation is complex [44, 45]. In this study, our assessment of “cerebral ischemia” was limited to radiographic evidence based on searching clinical note text; we did not directly assess the presence of delayed cerebral ischemia (DCI) as defined by Vergouwen et al. [46]. Interestingly, the study by Gonzalez Gomez et al. [42] found that eosinophil levels were elevated in patients with SAH with poor functional outcome (mRS), but appeared unrelated to DCI. Thus, it remains unclear whether the true treatable risk factor is DCI causing inflammation or if it is the inflammation itself, which could suggest an underlying epileptogenic process. Further, our results did not reproduce previous findings indicating that vasospasm and cerebral ischemia are associated with post-SAH epilepsy [2, 4, 41].

Compared with peripheral measures, CSF inflammatory markers are closer indicators of the degree of inflammation in the brain. Elevated CSF inflammatory markers after SAH have been linked to the development of complications such as hydrocephalus [47] as well as worse functional outcome [11] following SAH, but their relationship to post-SAH epilepsy remains underexplored. We found that delayed elevation of CSF WBCs were associated with the development of post-SAH epilepsy. Following SAH, CSF WBCs produce inflammatory cytokines such as intereukin-6 [47, 48] which has been shown to be elevated in patients with seizures [15] and which some evidence suggests plays a role in generating seizure activity [18]. RBCs present in CSF are broken down by phagocytes and release by-products such as hemoglobin which can trigger a neurotoxic inflammatory response that includes cytokine release and microglial activation [48] as well as upregulation of inflammasome proteins [11]. Multiple therapeutic strategies directed at reducing noxious blood products in CSF, including lumbar drainage and intrathecal haptoglobin infusion, have shown potential to improve clinical outcomes after SAH [10]. Thus, persistently elevated CSF WBC and RBC could increase the risk of post-SAH epilepsy by contributing to a sustained proinflammatory state that is neurotoxic and increases the likelihood of seizure generation.

### The Role of Blood Location

This is the first study to apply an anatomically directed longitudinal image analysis pipeline approach to measure the extent and location of blood over time in patients with SAH. Although patients who developed epilepsy tended to have a greater burden of blood overall, persistence of blood in pericortical areas during the first week after SAH was identified as a strong potential risk factor. Overall, our results complement existing findings that reduced blood clearance is implicated in the development of post-SAH complications [49–51] and that blood localization is related to the development of delayed cerebral ischemia [7, 8]. Also consistent with previous work [2], we found that ICH on admission was associated with an increased risk of post-SAH epilepsy, regardless of how blood evolved over time.

Blood volume within the cisterns, where most blood typically collects after aneurysmal rupture, has a stronger relationship to delayed cerebral ischemia than blood in other locations [8]. However, we found that cisternal blood was unrelated to the risk of developing epilepsy. This was surprising, given that cisternal blood can lead to delayed cerebral ischemia through neurotoxic inflammation [29, 48]. Blood products in direct contact with cortex likely produce a similar neuroinflammatory state that ultimately leads to increased neuronal synchrony and the development of recurrent, spontaneous seizures. Therefore, treatments that reduce cortical exposure to sustained blood and blood products [52, 53] could be beneficial for preventing post-SAH epilepsy.

### Advantages and Limitations of this Approach

Considerable interest exists for the development of artificial intelligence tools to manage complex neurological patients [21]. Major obstacles include the variability in timing of when data is obtained as well as the number of data points for each variable. The findings presented here are the result of a multi-stage workflow which is capable of handling multi-modal time series data and that incorporates well-defined statistical tools as well as iterative manual review of the results. The stepwise approach involves screening with a curve fitting method (SSANOVA) followed by selection of variables using a well-established statistical modeling approach for repeated measures data (GEE). This enabled us to identify novel associations between expected and unexpected longitudinal variables and post-SAH epilepsy in a relatively small patient cohort. Validation in larger patient cohorts will be critical.

Our approach also enabled us to explore dynamic correlations among many different types of variables, as shown in Fig. 6. Uncovering these relationships offers the potential to discover important pathophysiological interactions. For example, our results suggest that higher blood pressure during days 7–13 following SAH could be related to elevated SII. Interestingly, SII has been positively associated with increased risk of hypertension in retrospective and prospective studies [54–56] and identified as a predictor of stroke risk in patients with hypertension [57]. Together with the result indicating that higher SII, particularly in days 10–13, is associated with post-SAH epilepsy, this raises the possibility that closer blood pressure management could lessen the risk of developing epilepsy. The correlation analysis demonstrates the utility of our approach to extract unexpected relationships between variables and identify prospectively testable clinical interventions that could improve outcomes.

A major goal of this project was to develop a combined imaging and clinical longitudinal data workflow to identify potential risk factors for post-SAH epilepsy. As such, this work is only hypothesis-generating and has important limitations. We were only able to contact and obtain consent from a relatively small subset of the patients who met study criteria in phase 1. Of note, the coronavirus disease-19 (COVID-19) pandemic-related shutdown, together with existing structural and economic barriers faced many of our patients, likely affected our ability to reach many patients by phone. This impacted our sample size and may have introduced enrollment bias, given that a subset of patients who could not respond to the phase 2 survey may have developed severe disability, neurologic or otherwise, and potentially a higher burden of epilepsy. Our small sample size and the variability in the number of available retrospective datapoints across variables and patients further limits our sensitivity for some variables and will enable detection of only the most significant, time-dependent changes. The variability in data density across patients also limited our time resolution ultimately to 48-h (at minimum) windows. In addition, the distribution of SAH blood is highly variable within and across patients, making it difficult not only for trained radiologists but especially for machine learning methods to distinguish reliably [25, 58]. Though we used an established, validated SAH segmentation algorithm [26], we observed marked intra- and inter-patient variation in SAH detection. There is still substantial room for improvement in automated SAH detection, and future work could aim to develop and implement more reliable algorithms which are critical for making large-scale studies similar to ours tractable. We had relatively few patients with serial CSF measures; however, our results suggest CSF inflammation should be studied further. It will be important in future studies to focus on subpopulations of patients with nontraumatic SAH due to the same etiology to ensure differences in pathophysiology do not confound results. The potential effect of residual intravenous angiography contrast is a technical issue with the CT analysis that should be investigated further in future work. Finally, we lacked sufficient sample size to isolate separate training and testing cohorts or do cross-validation for the logistic regression modeling. Therefore, we do not claim to have identified definitive predictors of post-SAH epilepsy; we only suggest that the influential factors we did explore and identify could point in a new promising direction and inform future work. For instance, SII was highly associated with post-SAH epilepsy via our two-step statistical approach, and it appeared in nearly all the highest-performing exploratory models. Based on these findings, we argue only that SII, like other measures we identified and discuss here, may be worth further investigation as a marker of post-SAH epilepsy risk. To that end, our findings are preliminary and should be validated in larger cohorts. Ideally, we hope to use this approach in a larger, prospective cohort with serial imaging and clinical data collected consistently across all patients. This would allow validation of our current findings and the development of a robust assessment of clinically modifiable factors that may lead to post-SAH epilepsy.

### Conclusions

We demonstrated the utility of a multi-step approach that combines an automated image analysis pipeline with longitudinal neuro-ICU data to uncover time-dependent variables associated with the development of epilepsy after SAH. Our findings suggest elevated blood volumes early on, particularly in pericortical areas, and sustained inflammation at late time points could be important risk factors for post-SAH epilepsy and suggest that treatments which facilitate clearance of blood products from the CSF spaces and prevent sustained systemic and brain and peripheral inflammation could reduce the risk of post-SAH epilepsy. These results may not only inform future predictive models, but also identify high-risk patients for clinical trials aimed at preventing post-SAH epilepsy.

## Supplementary Information

Below is the link to the electronic supplementary material.

Supplementary file1 (DOCX 1598 KB)

## Data Availability

The data that support the findings of this study are available on request from the corresponding author. The data are not publicly available due to privacy or ethical restrictions.

## References

[1] Choi KS, Chun HJ, Yi HJ, Ko Y, Kim YS, Kim JM. Seizures and epilepsy following aneurysmal subarachnoid hemorrhage: incidence and risk factors. J Korean Neurosurg Soc. 2009;46(2):93–8. 10.3340/jkns.2009.46.2.93. 19763209 10.3340/jkns.2009.46.2.93 PMC2744032

[2] Huttunen J, Kurki MI, von und zu Fraunberg M, et al. Epilepsy after aneurysmal subarachnoid hemorrhage: a population-based, long-term follow-up study. Neurology. 2015;84(22):2229–37. 10.1212/WNL.0000000000001643. 25948726 10.1212/WNL.0000000000001643

[3] Oppong MD, Lohrer L, Wrede KH, et al. Reevaluation of risk factors for aneurysmal subarachnoid hemorrhage associated epilepsy. J Neurol Sci. 2023. 10.1016/j.jns.2022.120519. 10.1016/j.jns.2022.120519 36563606

[4] Nakashima S, Nishibayashi H, Yako R, et al. Factors associated with early and late seizure related to aneurysmal subarachnoid hemorrhage. Neurol Med Chir(Tokyo). 2024. 10.2176/jns-nmc.2023-0201. 38296550 10.2176/jns-nmc.2023-0201 PMC10992983

[5] Darkwah Oppong M, Lohrer L, Wrede KH, et al. Reevaluation of risk factors for aneurysmal subarachnoid hemorrhage associated epilepsy. J Neurol Sci. 2023;444:120519. 10.1016/j.jns.2022.12051. 36563606 10.1016/j.jns.2022.120519

[6] Hart Y, Sneade M, Birks J, Rischmiller J, Kerr R, Molyneux A. Epilepsy after subarachnoid hemorrhage: the frequency of seizures after clip occlusion or coil embolization of a ruptured cerebral aneurysm: results from the International Subarachnoid Aneurysm Trial. J Neurosurg. 2011;115(6):1159–68. 10.3171/2011.6.JNS101836. 21819189 10.3171/2011.6.JNS101836

[7] Yuan JY, Chen Y, Jayaraman K, et al. Automated quantification of compartmental blood volumes enables prediction of delayed cerebral ischemia and outcomes after aneurysmal subarachnoid hemorrhage. World Neurosurg. 2023;170:e214–22. 10.1016/j.wneu.2022.10.105. 36323345 10.1016/j.wneu.2022.10.105 PMC10995956

[8] van der Steen WE, Zijlstra IA, Verbaan D, et al. Association of quantified location-specific blood volumes with delayed cerebral ischemia after aneurysmal subarachnoid hemorrhage. AJNR Am J Neuroradiol. 2018;39(6):1059–64. 10.3174/ajnr.A5626. 29650786 10.3174/ajnr.A5626 PMC7410623

[9] Haapaniemi E, Strbian D, Rossi C, et al. The CAVE score for predicting late seizures after intracerebral hemorrhage. Stroke. 2014;45(7):1971–6. 10.1161/STROKEAHA.114.004686. 24876089 10.1161/STROKEAHA.114.004686

[10] Bandyopadhyay S, Schwendinger N, Jahromi BR, et al. Red blood cells in the cerebrospinal fluid compartment after subarachnoid haemorrhage: significance and emerging therapeutic strategies. Transl Stroke Res. 2024. 10.1007/s12975-024-01238-9. 38418755 10.1007/s12975-024-01238-9 PMC11772394

[11] Hirsch Y, Geraghty JR, Katz EA, Testai FD. Inflammasome Caspase-1 activity is elevated in cerebrospinal fluid after aneurysmal subarachnoid hemorrhage and predicts functional outcome. Neurocrit Care. 2021;34(3):889–98. 10.1007/s12028-020-01113-z. 32996055 10.1007/s12028-020-01113-z PMC8007683

[12] Nadkarni NA, Maas MB, Batra A, et al. Elevated cerebrospinal fluid protein is associated with unfavorable functional outcome in spontaneous subarachnoid hemorrhage. J Stroke Cerebrovasc Dis. 2020;29(4):104605. 10.1016/j.jstrokecerebrovasdis.2019.104605. 31932209 10.1016/j.jstrokecerebrovasdis.2019.104605 PMC7066873

[13] Cai L, Zeng H, Tan X, Wu X, Qian C, Chen G. The role of the blood neutrophil-to-lymphocyte ratio in aneurysmal subarachnoid hemorrhage. Front Neurol. 2021. 10.3389/fneur.2021.671098. 34149601 10.3389/fneur.2021.671098 PMC8209292

[14] Feghali J, Kim J, Gami A, et al. Monocyte-based inflammatory indices predict outcomes following aneurysmal subarachnoid hemorrhage. Neurosurg Rev. 2021;44(6):3499–507. 10.1007/s10143-021-01525-1. 33839947 10.1007/s10143-021-01525-1

[15] Aronica E, Crino PB. Inflammation in epilepsy: clinical observations. Epilepsia. 2011;52(s3):26–32. 10.1111/j.1528-1167.2011.03033.x. 21542843 10.1111/j.1528-1167.2011.03033.x

[16] Lissak IA, Zafar SF, Westover MB, et al. Soluble ST2 is associated with new epileptiform abnormalities following nontraumatic subarachnoid hemorrhage. Stroke. 2020;51(4):1128–34. 10.1161/STROKEAHA.119.028515. 32156203 10.1161/STROKEAHA.119.028515 PMC7123848

[17] Vezzani A. Epilepsy and inflammation in the brain: overview and pathophysiology: epilepsy and inflammation in the brain. Epilepsy Curr. 2014;14(2_suppl):3–7. 10.5698/1535-7511-14.s2.3. 24955068 10.5698/1535-7511-14.s2.3 PMC3966641

[18] Vezzani A, French J, Bartfai T, Baram TZ. The role of inflammation in epilepsy. Nat Rev Neurol. 2011;7(1):31–40. 10.1038/nrneurol.2010.178. 21135885 10.1038/nrneurol.2010.178 PMC3378051

[19] Huang Q, Zhang Z, Fan R, Liu S, Zheng W, Xiao F. Association of blood count–derived immunoinflammatory makers and risk of epilepsy: a prospective cohort of 497,291 participants. Seizure. 2024;123:9–16. 10.1016/j.seizure.2024.10.006. 39433008 10.1016/j.seizure.2024.10.006

[20] Geraghty JRB, Lung TJ, Hirsch Y, et al. Systemic immune-inflammation index predicts delayed cerebral vasospasm after aneurysmal subarachnoid hemorrhage. Neurosurgery. 2021;89(6):1071–9. 10.1093/neuros/nyab354. 34560777 10.1093/neuros/nyab354 PMC8600162

[21] Alkhachroum A, Terilli K, Megjhani M, Park S. Harnessing big data in neurocritical care in the era of precision medicine. Curr Treat Options Neurol. 2020;22(5):15. 10.1007/s11940-020-00622-8.

[22] Fisher RS, Acevedo C, Arzimanoglou A, et al. ILAE official report: a practical clinical definition of epilepsy. Epilepsia. 2014;55(4):475–82. 10.1111/epi.12550. 24730690 10.1111/epi.12550

[23] Marder CP, Narla V, Fink JR, Tozer Fink KR. Subarachnoid hemorrhage: beyond aneurysms. AJR Am J Roentgenol. 2014;202(1):25–37. 10.2214/AJR.12.9749. 24370126 10.2214/AJR.12.9749

[24] Maharathi B, Mir F, Hosur K, Loeb JA. INTUITION: a data platform to integrate human epilepsy clinical care and support for discovery. Front Digit Health. 2023. 10.3389/fdgth.2023.1091508. 37363274 10.3389/fdgth.2023.1091508 PMC10285513

[25] Butler M, Shah P, Ozgen B, et al. Automated segmentation of ventricular volumes and subarachnoid hemorrhage from computed tomography images: evaluation of a rule-based pipeline approach. Neuroradiol J. 2024. 10.1177/19714009241260791. 38869365 10.1177/19714009241260791 PMC11571338

[26] Thanellas A, Peura H, Lavinto M, et al. Development and external validation of a deep learning algorithm to identify and localize subarachnoid hemorrhage on CT scans. *Neurology*. Published online January 13, 2023. 10.1212/WNL.0000000000201710 10.1212/WNL.0000000000201710 PMC10033159 36639236

[27] Zhang J, Jin H, Wang Y, Sun X, Ma P, Zhong W. Smoothingspline ANOVA models and their applications in complex and massive datasets. In: Truong YKN, Sarfraz M, eds. *Topics in Splines and Applications*. InTech; 2018. 10.5772/intechopen.75861

[28] Cao T, Reeder HT, Foulkes AS. Functional principal component analysis and sparse-group LASSO to identify associations between biomarker trajectories and mortality among hospitalized SARS-CoV-2 infected individuals. BMC Med Res Methodol. 2023;23(1):254. 10.1186/s12874-023-02076-3. 37898791 10.1186/s12874-023-02076-3 PMC10613396

[29] Geraghty JR, Testai FD. Delayed cerebral ischemia after subarachnoid hemorrhage: beyond vasospasm and towards a multifactorial pathophysiology. Curr Atheroscler Rep. 2017;19(12):50. 10.1007/s11883-017-0690-x. 29063300 10.1007/s11883-017-0690-x

[30] Fujii M, Yan J, Rolland WB, Soejima Y, Caner B, Zhang JH. Early brain injury, an evolving frontier in subarachnoid hemorrhage research. Transl Stroke Res. 2013;4(4):432–46. 10.1007/s12975-013-0257-2. 23894255 10.1007/s12975-013-0257-2 PMC3719879

[31] Hofmann BB, Donaldson DM, Neyazi M, et al. Clinical outcome prediction of early brain injury in aneurysmal subarachnoid hemorrhage: the SHELTER-Score. Neurocrit Care. 2023. 10.1007/s12028-023-01879-y. 38030877 10.1007/s12028-023-01879-y PMC10959788

[32] Malinova V, Kranawetter B, Tuzi S, Moerer O, Rohde V, Mielke D. Interaction of optimal cerebral perfusion pressure with early brain injury and its impact on ischemic complications and outcome following aneurysmal subarachnoid hemorrhage. Neurocrit Care. 2023. 10.1007/s12028-023-01822-1. 37726549 10.1007/s12028-023-01822-1 PMC11147945

[33] Freiman S, Hauser WA, Rider F, Gulyaeva N, Guekht A. Post-stroke epilepsy: from clinical predictors to possible mechanisms. Epilepsy Res. 2024;199:107282. 10.1016/j.eplepsyres.2023.107282. 38134643 10.1016/j.eplepsyres.2023.107282

[34] R Core Team. *R: A Language and Environment for Statistical Computing*. R Foundation for Statistical Computing; 2023. https://www.R-project.org/

[35] Heymans M. *Psfmi: Prediction model pooling, selection and performance evaluation across multiply imputed datasets*.; 2023. https://CRAN.R-project.org/package=psfmi

[36] Wei T, Simko V. *R Package “Corrplot”: visualization of a correlation matrix*.; 2021. https://github.com/taiyun/corrplot

[37] Geraghty JR, Butler M, Maharathi B, et al. Diffuse microglial responses and persistent EEG changes correlate with poor neurological outcome in a model of subarachnoid hemorrhage. Sci Rep. 2024;14(1):13618. 10.1038/s41598-024-64631-2. 38871799 10.1038/s41598-024-64631-2 PMC11176397

[38] Yi HJ, Shin DS, Kim BT. Dynamic changes of systemic inflammation response index and systemic immune-inflammation index are associated with delayed cerebral ischemia after aneurysmal subarachnoid hemorrhage. J Stroke Cerebrovasc Dis. 2024. 10.1016/j.jstrokecerebrovasdis.2024.107626. 38325674 10.1016/j.jstrokecerebrovasdis.2024.107626

[39] Jamali SA, Turnbull MT, Kanekiyo T, et al. Elevated neutrophil-lymphocyte ratio is predictive of poor outcomes following aneurysmal subarachnoid hemorrhage. J Stroke Cerebrovasc Dis. 2020;29(4):104631. 10.1016/j.jstrokecerebrovasdis.2019.104631. 31964576 10.1016/j.jstrokecerebrovasdis.2019.104631

[40] Ray B, Ross SR, Danala G, et al. Systemic response of coated-platelet and peripheral blood inflammatory cell indices after aneurysmal subarachnoid hemorrhage and long-term clinical outcome. J Crit Care. 2019;52:1–9. 10.1016/j.jcrc.2019.03.003. 30904732 10.1016/j.jcrc.2019.03.003 PMC8663918

[41] Claassen J, Peery S, Kreiter KT, et al. Predictors and clinical impact of epilepsy after subarachnoid hemorrhage. Neurology. 2003;60(2):208–14. 10.1212/01.WNL.0000038906.71394.DE. 12552032 10.1212/01.wnl.0000038906.71394.de

[42] Gonzalez Gomez H, Savarraj JPJ, Paz AS, et al. Peripheral eosinophil trends and clinical outcomes after non-traumatic subarachnoid hemorrhage. Front Neurol. 2023. 10.3389/fneur.2023.1051732. 36895904 10.3389/fneur.2023.1051732 PMC9989180

[43] Marchi N, Granata T, Ghosh C, Janigro D. Blood–brain barrier dysfunction and epilepsy: pathophysiologic role and therapeutic approaches. Epilepsia. 2012;53(11):1877–86. 10.1111/j.1528-1167.2012.03637.x. 22905812 10.1111/j.1528-1167.2012.03637.x PMC4842020

[44] Al-Tamimi YZ, Bhargava D, Orsi NM, et al. Compartmentalisation of the inflammatory response following aneurysmal subarachnoid haemorrhage. Cytokine. 2019;123:154778. 10.1016/j.cyto.2019.154778. 31323526 10.1016/j.cyto.2019.154778

[45] Mohme M, Sauvigny T, Mader MMD, et al. Immune characterization in aneurysmal subarachnoid hemorrhage reveals distinct monocytic activation and chemokine patterns. Transl Stroke Res. 2020;11(6):1348–61. 10.1007/s12975-019-00764-1. 31858408 10.1007/s12975-019-00764-1

[46] Vergouwen MDI, Vermeulen M, van Gijn J, et al. Definition of delayed cerebral ischemia after aneurysmal subarachnoid hemorrhage as an outcome event in clinical trials and observational studies. Stroke. 2010;41(10):2391–5. 10.1161/STROKEAHA.110.589275. 20798370 10.1161/STROKEAHA.110.589275

[47] Takizawa T, Tada T, Kitazawa K, et al. Inflammatory cytokine cascade released by leukocytes in cerebrospinal fluid after subarachnoid hemorrhage. Neurol Res. 2001;23(7):724–30. 10.1179/016164101101199243. 11680512 10.1179/016164101101199243

[48] Geraghty JR, Davis JL, Testai FD. Neuroinflammation and microvascular dysfunction after experimental subarachnoid hemorrhage: emerging components of early brain injury related to outcome. Neurocrit Care. 2019;31(2):373–89. 10.1007/s12028-019-00710-x. 31012056 10.1007/s12028-019-00710-x PMC6759381

[49] Zeineddine HA, Divito A, McBride DW, et al. Subarachnoid blood clearance and aneurysmal subarachnoid hemorrhage outcomes: a retrospective review. Neurocrit Care. 2023. 10.1007/s12028-023-01729-x. 37100974 10.1007/s12028-023-01729-x

[50] Reilly C, Amidei C, Tolentino J, Jahromi BS, Macdonald RL. Clot volume and clearance rate as independent predictors of vasospasm after aneurysmal subarachnoid hemorrhage. J Neurosurg. 2004;101(2):255–61. 10.3171/jns.2004.101.2.0255. 15309916 10.3171/jns.2004.101.2.0255

[51] Ritzenthaler T, Gobert F, Bouchier B, Dailler F. Amount of blood during the subacute phase and clot clearance rate as prognostic factors for delayed cerebral ischemia after aneurysmal subarachnoid hemorrhage. J Clin Neurosci. 2021;87:74–9. 10.1016/j.jocn.2021.02.007. 33863538 10.1016/j.jocn.2021.02.007

[52] Florez-Perdomo W, Mishra R, García-Ballestas E, et al. Cisternal irrigation and clot removal to prevent vasospasm and poor outcome in aneurysmal subarachnoid hemorrhage: systematic review and meta-analysis. Int J Surg Open. 2022;43:100459. 10.1016/j.ijso.2022.100459.

[53] Blackburn SL, Grande AW, Swisher CB, Hauck EF, Jagadeesan B, Provencio JJ. Prospective trial of cerebrospinal fluid filtration after aneurysmal subarachnoid hemorrhage via lumbar catheter (PILLAR). Stroke. 2019;50(9):2558–61. 10.1161/STROKEAHA.119.025399. 31345133 10.1161/STROKEAHA.119.025399 PMC6710124

[54] Chen Y, Li Y, Liu M, Xu W, Tong S, Liu K. Association between systemic immunity-inflammation index and hypertension in US adults from NHANES 1999-2018. Sci Rep. 2024;14(1):5677. 10.1038/s41598-024-56387-6. 38454104 10.1038/s41598-024-56387-6 PMC10920861

[55] Ma LL, Xiao HB, Zhang J, et al. Association between systemic immune inflammatory/inflammatory response index and hypertension: a cohort study of functional community. Nutr Metab Cardiovasc Dis. 2024;34(2):334–42. 10.1016/j.numecd.2023.09.025. 38000992 10.1016/j.numecd.2023.09.025

[56] Xu JP, Zeng RX, Zhang YZ, et al. Systemic inflammation markers and the prevalence of hypertension: a NHANES cross-sectional study. Hypertens Res. 2023;46(4):1009–19. 10.1038/s41440-023-01195-0. 36707716 10.1038/s41440-023-01195-0

[57] Aydin C, Alpsoy Ş, Akyüz A, et al. Could the systemic immune-inflammation index be a predictor to estimate cerebrovascular events in hypertensive patients? Blood Press Monit. 2022;27(1):33–8. 10.1097/MBP.0000000000000560. 34992205 10.1097/MBP.0000000000000560

[58] Hu P, Zhou H, Yan T, et al. Deep learning-assisted identification and quantification of aneurysmal subarachnoid hemorrhage in non-contrast CT scans: development and external validation of hybrid 2D/3D UNet. Neuroimage. 2023. 10.1016/j.neuroimage.2023.120321. 37574119 10.1016/j.neuroimage.2023.120321

