# Supplementary material for: A Longitudinal Imaging and Clinical Data Workflow Identifies Potential Time-Dependent Risk Factors for Post-Subarachnoid Hemorrhage Epilepsy
Source: Neurocrit Care. 2026 Mar 20;45(1):351–67. doi: 10.1007/s12028-026-02482-7 (PMC13369638; doi:10.1007/s12028-026-02482-7)
Supplement: Supplementary file 1 — Supplementary file1 (DOCX 1598 KB) [file 12028_2026_2482_MOESM1_ESM.docx]

**SUPPLEMENTARY DATA**

**
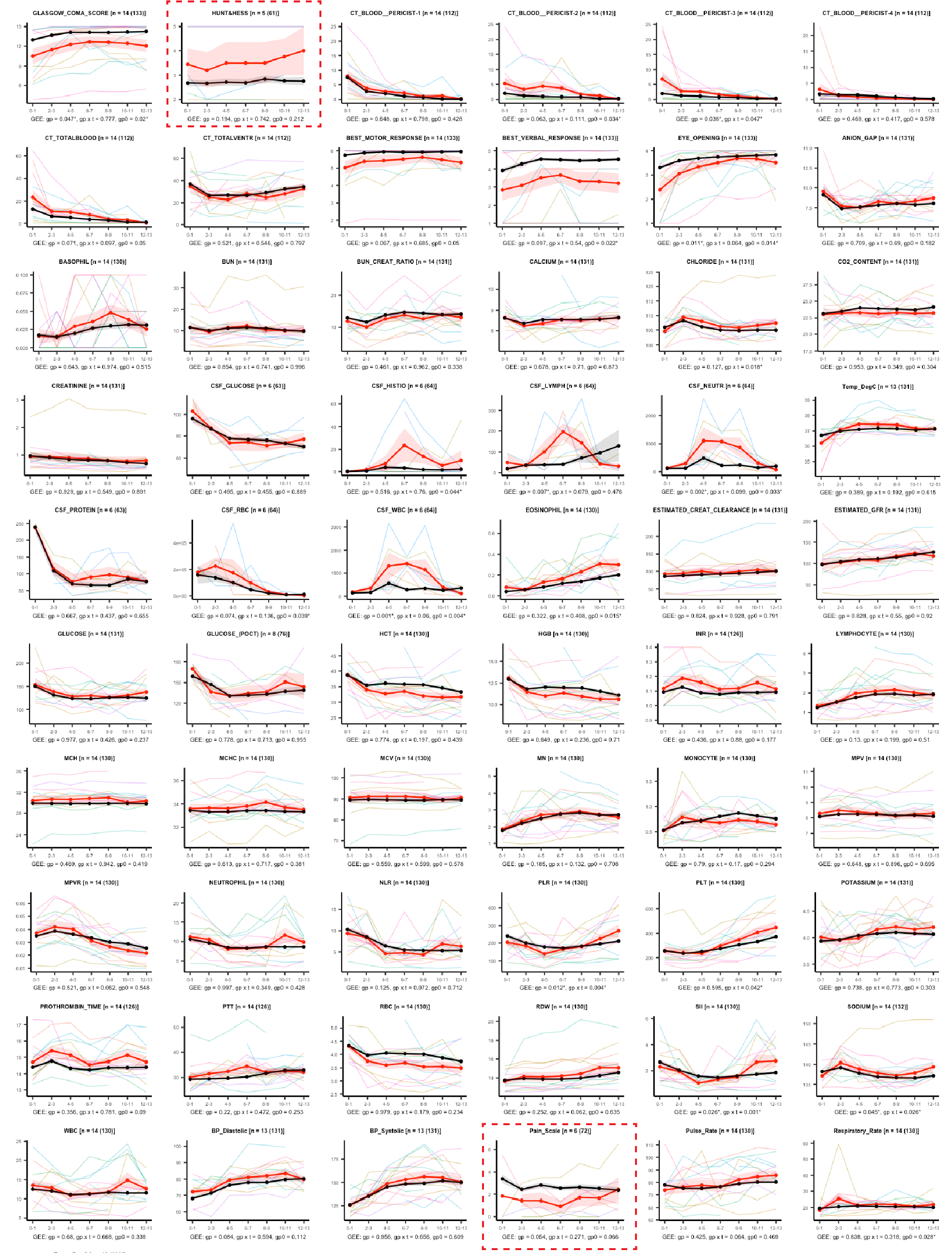
**

**Figure S1. Time series curves for all longitudinal variables analyzed in the study.** Hunt & Hess Score and Pain Scale (red dashed boxes) were collected serially for a relatively small number of patients and were analyzed together variables in our pipeline. However, they were not considered as major longitudinal variables of interest in this study. Hunt&Hess Score from admission were used to define “low-grade” vs “high-grade” SAH (see Table 1). Selected GEE p-values are shown at the bottom of each plot: “gp0” denotes additive model group p-value; “gp” denotes interaction model group p-value; “gp x t” denotes group-by-time interaction p-value. For some variables with group-by-time interaction p-value <0.05, the “gp0” values are hidden here. See Table S1 for complete results from SSANOVA and GEE analysis. Cell counts are displayed as: [1000 cells / µL ] for serum variables, [cells / µL] for CSF variables.

**
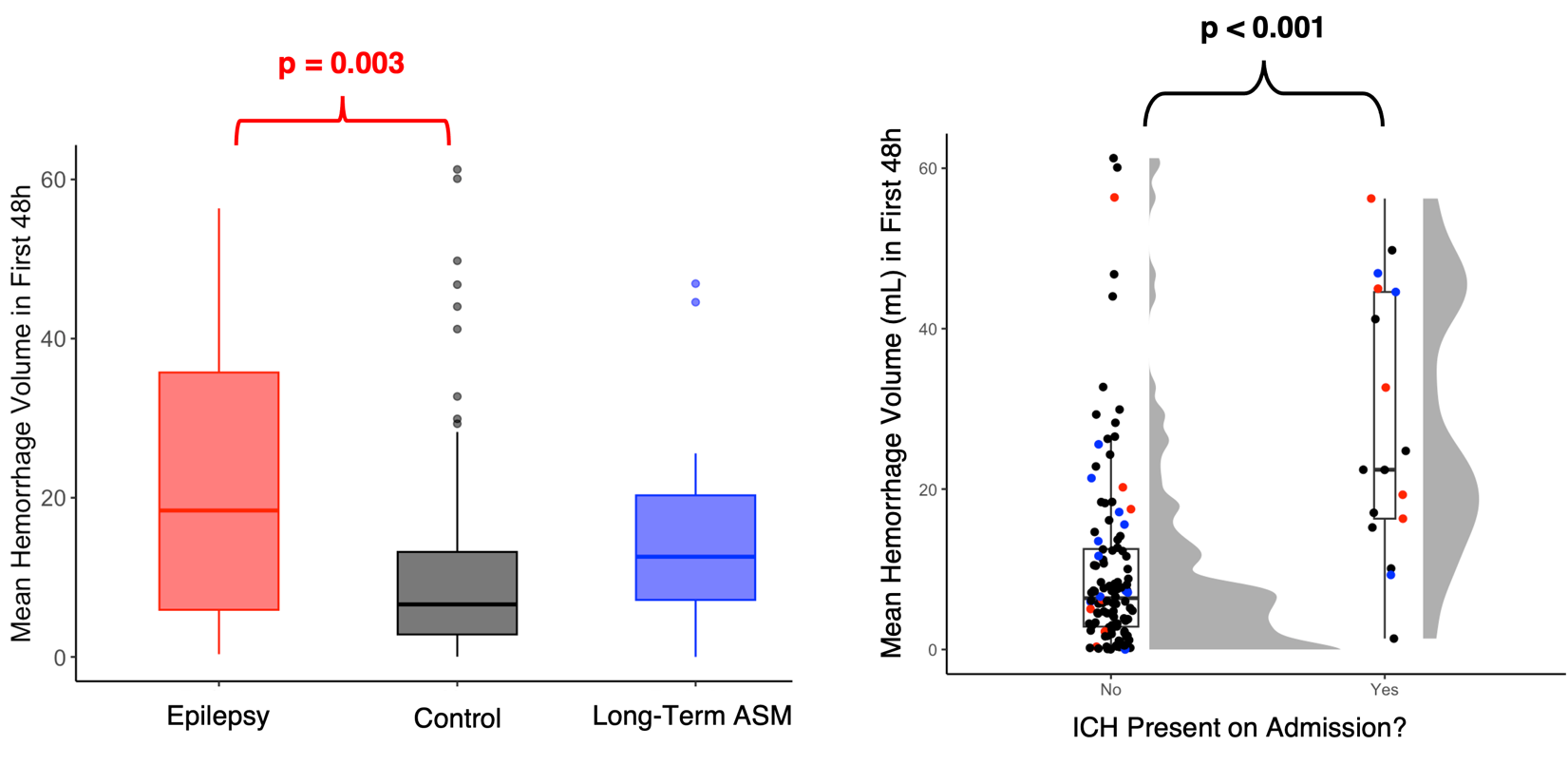
**

**Figure S2. Presence of ICH on admission is associated with larger blood volumes in the first 48h after SAH as well as the development of post-SAH epilepsy.** Taken together with the time series curves in Figure 3, these results suggest that the longitudinal effect of parenchymal blood volume on post-SAH epilepsy, as identified by GEE analysis, can be effectively captured by overall blood volume and presence of ICH on admission. Patients who did not meet criteria for post-SAH epilepsy diagnosis but were on long-term anti-seizure medications (ASMs) when contacted (“Long-Term ASM” group) are included for reference. [Left] Mean hemorrhage volume in the first 48h following SAH stratified by patient group. Total hemorrhage volumes were significantly larger for epilepsy patients (red) vs controls (black). [Right] Patients with ICH on admission had significantly higher blood volumes in the first 48h following SAH. Kruskall-Wallis test was used to assess for differences between groups. For the plot on the Left, Kruskall-Wallis was followed by Wilcoxon rank sum test for pairwise comparisons with Bonferroni correction.

**Table S1. SAH etiologies for patients who developed epilepsy.** For each patient who did not have aneurysmal SAH source (n=3), a note on the etiology based on manual chart review is shown.

| **patient** | **aneurysm** | **sah_type_note** |
| --- | --- | --- |
| 1 | Yes | large 7 x 3 cm right frontal ICH with IVH and SAH |
| 2 | No | **No identifiable source on MRI or CTA.** Dx = "Left cortical subarachnoid"; suspected gyriform hyperdenisities in the left superior forntal lobe suspicious for subarachnoid hemorrhage w/ underlying local mass effect. |
| 3 | Yes |  |
| 4 | Yes |  |
| 5 | Yes |  |
| 6 | Yes |  |
| 7 | Yes |  |
| 8 | Yes |  |
| 9 | Yes |  |
| 10 | No | per DC Summary: "R high convexity frontoparietal intraparenchymal hemorrhage measuring 3x2cm along w/ a R sylvian fissure SAH as confirmed on CT by OSH", "Angio showed **pial AVF/AVM fed by PCA** which may have resulted in venous hypertension and reflux causing parietal lobe hemorrhage. No other vascular malformations or aneurysms were evident." |
| 11 | Yes |  |
| 12 | Yes |  |
| 13 | Yes |  |
| 14 | Yes |  |
| 15 | No | IPH; Right-sided temporoparietal intraparenchymal hemorrhage with mass effect on brain and midline shift, **secondary to ruptured arteriovenous malformation** |

**Table S2. Summary statistics and results for two-stage SSANOVA + GEE analysis of longitudinal variables.**

|  | **SSANOVA** | **GEE** | | |  |
| --- | --- | --- | --- | --- | --- |
| **Variable** | **Range of Days with Group Difference** | **Group *P*-value**  **(Interaction Model)** | **Group x Time Interaction *P*-value** | **Group *P*-value (Additive Model)** | **Type** |
| GCS | 0-13 | **0.047*^a^*** | 0.777 | **0.020** | Clinical |
| GCS Motor | 0-13 | 0.067 | 0.685 | 0.050* | Clinical |
| GCS Verbal | 0-13 | 0.097 | 0.540 | **0.022** | Clinical |
| GCS Eye Opening | 0-13 | **0.011*^a^*** | 0.064 | **0.014** | Clinical |
| Diastolic BP |  | 0.084 | 0.594 | 0.112 | Clinical |
| Systolic BP |  | 0.956 | 0.656 | 0.609 | Clinical |
| Heart Rate |  | 0.425 | 0.064 | 0.469 | Clinical |
| Respiratory Rate |  | 0.838 | 0.318 | **0.028*^b^*** | Clinical |
| Temperature (Celsius) |  | 0.389 | 0.192 | 0.615 | Clinical |
| Pericortical Blood Volume | 0-9 | 0.063 | 0.111 | **0.034** | Imaging |
| Parenchymal Blood Volume *^c^* | 0-5 | **0.038** | **0.047** | **0.035** | Imaging |
| Total Blood Volume | 0-8 | 0.071 | 0.097 | 0.050* | Imaging |
| Cisternal Blood Volume |  | 0.648 | 0.798 | 0.428 | Imaging |
| Periventricular Blood Volume |  | 0.469 | 0.417 | 0.578 | Imaging |
| Lateral Ventricle Volume |  | 0.521 | 0.546 | 0.797 | Imaging |
| Hematocrit | 3-13 | 0.774 | 0.197 | 0.439 | Hematologic |
| Hemoglobin | 4-13 | 0.649 | 0.236 | 0.710 | Hematologic |
| MCH | 4-9 | 0.469 | 0.942 | 0.419 | Hematologic |
| MCHC | 5-11 | 0.613 | 0.717 | 0.381 | Hematologic |
| Eosinophils | 2-13 | **0.322** | **0.408** | **0.015** | Hematologic |
| Monocytes | 10-13 | 0.790 | 0.170 | 0.294 | Hematologic |
| Neutrophils | 11-13 | 0.997 | 0.349 | 0.428 | Hematologic |
| Red Blood Cells | 3-13 | 0.979 | 0.179 | 0.234 | Hematologic |
| RDW | 7-13 | 0.252 | 0.062 | 0.635 | Hematologic |
| SII | 10-13 | **0.026** | **0.001** | 0.671 | Hematologic |
| Basophil |  | 0.643 | 0.974 | 0.515 | Hematologic |
| Lymphocytes |  | 0.130 | 0.199 | 0.510 | Hematologic |
| MCV |  | 0.559 | 0.599 | 0.578 | Hematologic |
| MN |  | 0.185 | 0.132 | 0.708 | Hematologic |
| NLR |  | 0.125 | 0.072 | 0.712 | Hematologic |
| PLR |  | **0.012** | **0.004** | 0.938 | Hematologic |
| White Blood Cells |  | 0.680 | 0.668 | 0.338 | Hematologic |
| INR | 2-9 | 0.436 | 0.880 | 0.177 | Coagulation |
| PLT | 6-13 | 0.595 | **0.042** | 0.183 | Coagulation |
| Prothrombin Time | 1-11 | 0.356 | 0.781 | 0.090 | Coagulation |
| MPV |  | 0.648 | 0.896 | 0.695 | Coagulation |
| MPVR |  | 0.521 | 0.062 | 0.548 | Coagulation |
| PTT |  | 0.220 | 0.472 | 0.253 | Coagulation |
| Chloride | 7-13 | 0.127 | **0.018** | 0.502 | Metabolic |
| Potassium | 9-11 | 0.738 | 0.773 | 0.303 | Metabolic |
| Sodium | 7-13 | **0.045*^b^*** | **0.026** | 0.338 | Metabolic |
| Anion Gap |  | 0.709 | 0.690 | 0.182 | Metabolic |
| BUN |  | 0.854 | 0.741 | 0.996 | Metabolic |
| BUN/Creatinine |  | 0.461 | 0.962 | 0.338 | Metabolic |
| Calcium |  | 0.678 | 0.710 | 0.873 | Metabolic |
| CO2 |  | 0.953 | 0.349 | 0.304 | Metabolic |
| Creatinine |  | 0.929 | 0.549 | 0.891 | Metabolic |
| Est. Creatinine Clearance |  | 0.824 | 0.928 | 0.791 | Metabolic |
| Est. GFR |  | 0.828 | 0.550 | 0.920 | Metabolic |
| Glucose |  | 0.977 | 0.426 | 0.237 | Metabolic |
| Glucose (Point of Care Test) |  | 0.778 | 0.713 | 0.955 | Metabolic |
| CSF Histiocytes *^c^* | 4-13 | 0.516 | 0.760 | **0.044** | CSF |
| CSF Neutrophils | 3-11 | **0.002*^a^*** | 0.099 | **0.003** | CSF |
| CSF RBC | 2-5 | 0.074 | 0.136 | **0.039** | CSF |
| CSF WBC | 3-10 | **0.001*^a^*** | 0.060 | **0.004** | CSF |
| CSF Glucose |  | 0.495 | 0.455 | 0.889 | CSF |
| CSF Lymphocytes |  | **0.007*^a^*** | 0.079 | 0.476 | CSF |
| CSF Protein |  | 0.667 | 0.437 | 0.655 | CSF |

P-values <0.05 are bolded. With a few exceptions, variables were considered “selected” by GEE analysis if group-effect p-value was <0.05. GEE interaction model results were only used if the group-by-time interaction p-value was <0.05. For variables that passed SSANOVA screening, column 2 shows the range of Days (post-SAH) during which a substantial group-effect was observed. This provided a rough approximation of which time windows might be important for each potential variable of interest.

*^a^* Interaction model p-value for group effect <0.05 but group-x-time interaction p-value >0.05. In these cases, variables were only considered “selected” and included in further analysis if the additive model p-value <0.05.

*^b^* For each of these variables, manual review of group-averaged and individual epilepsy patient time series curves (see Figure S1) showed that a single value in a single patient was the driver of the group effect. These were therefore excluded from further analysis.

*^c^* We ultimately did not include parenchymal blood volume or CSF histiocytes in the exploratory logistic regression analysis. Histiocytes are infrequently measured in clinical CSF samples; measurements of this population in our samples showed a very low dynamic range (0-60 cells/µL); and the effect appeared to be driven by 1-2 patients (see Figure S1). The parenchymal blood volume effect, which extends at most to Day 5 (per SSANOVA) but appears predominantly isolated to the first 48h after SAH (see Figure S1), is effectively captured by the presence or absence of ICH on admission together with the total blood volume (on admission and over time). See Table 1, Figure S2. Total blood volume, with borderline group p-value=0.05, was thus included in our exploratory modeling analysis rather than parenchymal volume.

**Table S3. Ranked list of variables incorporated into our data-driven logistic regression analysis.**

| **Variable** | **Type** | **Correlate** |
| --- | --- | --- |
| CSF Neutrophils | CSF | Neuroinflammation |
| CSF WBC | CSF | Neuroinflammation |
| GCS Eye Opening | Clinical | Neurologic Deficit |
| Eosinophils | Hematologic | Systemic Inflammation |
| GCS | Clinical | Impaired Consciousness |
| GCS Verbal | Clinical | Impaired Consciousness |
| SII | Hematologic | Systemic Inflammation |
| Pericortical Blood Volume | Imaging | Brain Hemorrhage |
| CSF RBC | CSF | Neuroinflammation |
| Total Blood Volume | Imaging | Brain Hemorrhage |
| PLR | Hematologic | Systemic Inflammation |

Ranking is based on the additive model group p-value from GEE analysis (column 5 in Table S2 above), which we used as an initial proxy measure of strength of association with post-SAH epilepsy. Total blood volume and platelet-to-lymphocyte ratio (PLR) highlighted with light blue shading were included based on additional factors beyond GEE results (see Table S2), but both had group (additive OR interaction model) GEE p-values ≤0.05.

**Table S4. Significant pairwise correlations among longitudinal variables.**

| **varXY** | **win** | **r** | **lower.adj** | **upper.adj** | **p.adj** | **epi.assoc** |
| --- | --- | --- | --- | --- | --- | --- |
| best-motor-response__best-verbal-response | 1_3 | 0.715332 | 0.468094 | 0.85871 | 2.11E-16 | 1 |
| best-motor-response__best-verbal-response | 4_7 | 0.633272 | 0.363937 | 0.804876 | 4.63E-13 | 1 |
| best-motor-response__best-verbal-response | 8_14 | 0.547937 | 0.24231 | 0.754633 | 2.03E-08 | 1 |
| best-motor-response__eye-opening | 1_3 | 0.558215 | 0.235609 | 0.77001 | 1E-07 | 1 |
| best-motor-response__eye-opening | 4_7 | 0.427957 | 0.092123 | 0.676374 | 0.00044 | 1 |
| best-motor-response__glasgow-coma-score | 1_3 | 0.707853 | 0.45622 | 0.854665 | 7.35E-16 | 1 |
| best-motor-response__glasgow-coma-score | 4_7 | 0.551324 | 0.249611 | 0.755465 | 9.84E-09 | 1 |
| best-motor-response__glasgow-coma-score | 8_14 | 0.480627 | 0.154675 | 0.712219 | 9.97E-06 | 1 |
| best-motor-response__temp-degc | 8_14 | -0.36443 | -0.63456 | -0.01496 | 0.029051 | 0 |
| best-verbal-response__bp-systolic | 8_14 | -0.38765 | -0.65058 | -0.04174 | 0.007465 | 1 |
| best-verbal-response__eye-opening | 1_3 | 0.68368 | 0.41836 | 0.841496 | 3.23E-14 | 1 |
| best-verbal-response__eye-opening | 4_7 | 0.587559 | 0.299137 | 0.777607 | 1.7E-10 | 1 |
| best-verbal-response__eye-opening | 8_14 | 0.526215 | 0.213498 | 0.74112 | 1.74E-07 | 1 |
| best-verbal-response__glasgow-coma-score | 1_3 | 0.92504 | 0.843234 | 0.964968 | 6E-47 | 1 |
| best-verbal-response__glasgow-coma-score | 4_7 | 0.851641 | 0.714519 | 0.925753 | 2.36E-35 | 1 |
| best-verbal-response__glasgow-coma-score | 8_14 | 0.800977 | 0.624757 | 0.899557 | 2.6E-27 | 1 |
| best-verbal-response__inr | 4_7 | -0.38392 | -0.64742 | -0.03843 | 0.008696 | 1 |
| best-verbal-response__inr | 8_14 | -0.3927 | -0.65911 | -0.03871 | 0.009219 | 1 |
| best-verbal-response__neutrophil | 8_14 | -0.38086 | -0.64673 | -0.03245 | 0.012163 | 1 |
| best-verbal-response__nlr | 8_14 | -0.36242 | -0.63402 | -0.01122 | 0.034983 | 1 |
| best-verbal-response__pt | 4_7 | -0.35648 | -0.62835 | -0.00701 | 0.042463 | 1 |
| best-verbal-response__pt | 8_14 | -0.3742 | -0.64654 | -0.01727 | 0.026639 | 1 |
| best-verbal-response__sii | 4_7 | -0.35977 | -0.62983 | -0.0121 | 0.032964 | 1 |
| best-verbal-response__sii | 8_14 | -0.38258 | -0.64793 | -0.03441 | 0.010999 | 1 |
| best-verbal-response__sodium | 4_7 | -0.36139 | -0.63098 | -0.01393 | 0.030074 | 1 |
| best-verbal-response__temp-degc | 4_7 | -0.39371 | -0.65494 | -0.04843 | 0.005187 | 1 |
| best-verbal-response__temp-degc | 8_14 | -0.52871 | -0.74269 | -0.21677 | 1.37E-07 | 1 |
| blood-cisternal__best-verbal-response | 4_7 | -0.50184 | -0.73604 | -0.16034 | 1.31E-05 | 1 |
| blood-cisternal__best-verbal-response | 8_14 | -0.38266 | -0.66466 | -0.00522 | 0.048251 | 1 |
| blood-cisternal__blood-parenchymal | 1_3 | 0.700197 | 0.450756 | 0.848207 | 4.62E-16 | 1 |
| blood-cisternal__blood-parenchymal | 4_7 | 0.687767 | 0.424704 | 0.843734 | 1.75E-14 | 1 |
| blood-cisternal__blood-parenchymal | 8_14 | 0.704525 | 0.445273 | 0.854789 | 5.09E-15 | 1 |
| blood-cisternal__blood-pericortical | 1_3 | 0.579185 | 0.272316 | 0.779112 | 4.49E-09 | 1 |
| blood-cisternal__blood-pericortical | 4_7 | 0.630987 | 0.338874 | 0.812142 | 3.93E-11 | 1 |
| blood-cisternal__blood-pericortical | 8_14 | 0.637733 | 0.342541 | 0.818322 | 4.95E-11 | 1 |
| blood-cisternal__blood-periventricular | 1_3 | 0.52942 | 0.204641 | 0.74919 | 5.83E-07 | 0 |
| blood-cisternal__blood-periventricular | 4_7 | 0.552076 | 0.227235 | 0.766362 | 1.77E-07 | 0 |
| blood-cisternal__blood-periventricular | 8_14 | 0.5318 | 0.193091 | 0.757258 | 2.17E-06 | 0 |
| blood-cisternal__glasgow-coma-score | 4_7 | -0.40267 | -0.67318 | -0.03712 | 0.01066 | 1 |
| blood-cisternal__totalblood | 1_3 | 0.887606 | 0.773203 | 0.946056 | 5.34E-39 | 0 |
| blood-cisternal__totalblood | 4_7 | 0.856253 | 0.71068 | 0.931513 | 1.45E-31 | 0 |
| blood-cisternal__totalblood | 8_14 | 0.778903 | 0.568332 | 0.893717 | 5.08E-21 | 0 |
| blood-parenchymal__best-motor-response | 8_14 | -0.42862 | -0.69449 | -0.05976 | 0.003811 | 1 |
| blood-parenchymal__best-verbal-response | 1_3 | -0.49863 | -0.73406 | -0.15621 | 1.69E-05 | 1 |
| **varXY** | **win** | **r** | **lower.adj** | **upper.adj** | **p.adj** | **epi.assoc** |
| blood-parenchymal__best-verbal-response | 4_7 | -0.61352 | -0.80219 | -0.31345 | 3.1E-10 | 1 |
| blood-parenchymal__best-verbal-response | 8_14 | -0.58411 | -0.78861 | -0.26326 | 2.22E-08 | 1 |
| blood-parenchymal__blood-pericortical | 1_3 | 0.796917 | 0.609342 | 0.900022 | 8.03E-25 | 1 |
| blood-parenchymal__blood-pericortical | 4_7 | 0.7919 | 0.595428 | 0.898999 | 3.54E-23 | 1 |
| blood-parenchymal__blood-pericortical | 8_14 | 0.731832 | 0.48934 | 0.869281 | 5.46E-17 | 1 |
| blood-parenchymal__blood-periventricular | 1_3 | 0.741167 | 0.516046 | 0.870513 | 2.71E-19 | 1 |
| blood-parenchymal__blood-periventricular | 4_7 | 0.714079 | 0.466121 | 0.858025 | 2.6E-16 | 1 |
| blood-parenchymal__blood-periventricular | 8_14 | 0.710431 | 0.454692 | 0.857946 | 2E-15 | 1 |
| blood-parenchymal__csf-rbc | 8_14 | 0.496408 | 0.020034 | 0.789091 | 0.034729 | 1 |
| blood-parenchymal__eye-opening | 1_3 | -0.39148 | -0.6658 | -0.02392 | 0.020107 | 1 |
| blood-parenchymal__glasgow-coma-score | 1_3 | -0.4795 | -0.72221 | -0.13165 | 7.21E-05 | 1 |
| blood-parenchymal__glasgow-coma-score | 4_7 | -0.49939 | -0.73453 | -0.15717 | 1.59E-05 | 1 |
| blood-parenchymal__glasgow-coma-score | 8_14 | -0.52034 | -0.75117 | -0.17612 | 6.55E-06 | 1 |
| blood-parenchymal__inr | 8_14 | 0.395044 | 0.012132 | 0.676912 | 0.035935 | 1 |
| blood-parenchymal__nlr | 8_14 | 0.392175 | 0.016334 | 0.670912 | 0.029397 | 1 |
| blood-parenchymal__totalblood | 1_3 | 0.892648 | 0.782781 | 0.948549 | 3.97E-40 | 1 |
| blood-parenchymal__totalblood | 4_7 | 0.902258 | 0.798194 | 0.954031 | 1.31E-40 | 1 |
| blood-parenchymal__totalblood | 8_14 | 0.937199 | 0.865994 | 0.971153 | 2.23E-49 | 1 |
| blood-pericortical__best-motor-response | 1_3 | -0.37678 | -0.65605 | -0.00675 | 0.044492 | 1 |
| blood-pericortical__best-motor-response | 8_14 | -0.39357 | -0.67185 | -0.01793 | 0.027315 | 1 |
| blood-pericortical__best-verbal-response | 1_3 | -0.52574 | -0.75058 | -0.1918 | 1.85E-06 | 1 |
| blood-pericortical__best-verbal-response | 4_7 | -0.56388 | -0.77335 | -0.24342 | 5.78E-08 | 1 |
| blood-pericortical__best-verbal-response | 8_14 | -0.51626 | -0.74872 | -0.17072 | 9.06E-06 | 1 |
| blood-pericortical__blood-periventricular | 1_3 | 0.624117 | 0.336185 | 0.805351 | 2.57E-11 | 1 |
| blood-pericortical__blood-periventricular | 4_7 | 0.570786 | 0.252947 | 0.777421 | 2.94E-08 | 1 |
| blood-pericortical__blood-periventricular | 8_14 | 0.465267 | 0.106813 | 0.716689 | 0.00033 | 1 |
| blood-pericortical__eye-opening | 1_3 | -0.42529 | -0.68788 | -0.06424 | 0.002793 | 1 |
| blood-pericortical__glasgow-coma-score | 1_3 | -0.49347 | -0.73087 | -0.14955 | 2.52E-05 | 1 |
| blood-pericortical__glasgow-coma-score | 4_7 | -0.45654 | -0.70782 | -0.10269 | 0.000365 | 1 |
| blood-pericortical__glasgow-coma-score | 8_14 | -0.43159 | -0.69637 | -0.06337 | 0.00319 | 1 |
| blood-pericortical__totalblood | 1_3 | 0.779857 | 0.580214 | 0.891096 | 5.85E-23 | 1 |
| blood-pericortical__totalblood | 4_7 | 0.793012 | 0.597345 | 0.899573 | 2.69E-23 | 1 |
| blood-pericortical__totalblood | 8_14 | 0.855017 | 0.704844 | 0.931838 | 3.22E-30 | 1 |
| blood-periventricular__best-motor-response | 8_14 | -0.44343 | -0.70388 | -0.07788 | 0.001541 | 0 |
| blood-periventricular__best-verbal-response | 1_3 | -0.53736 | -0.75758 | -0.20731 | 6.75E-07 | 1 |
| blood-periventricular__best-verbal-response | 4_7 | -0.63097 | -0.81213 | -0.33885 | 3.94E-11 | 1 |
| blood-periventricular__best-verbal-response | 8_14 | -0.53267 | -0.75855 | -0.1925 | 2.39E-06 | 1 |
| blood-periventricular__csf-rbc | 8_14 | 0.533765 | 0.070499 | 0.807629 | 0.006152 | 1 |
| blood-periventricular__eye-opening | 1_3 | -0.41743 | -0.68279 | -0.05478 | 0.004499 | 1 |
| blood-periventricular__glasgow-coma-score | 1_3 | -0.49977 | -0.73476 | -0.15768 | 1.55E-05 | 1 |
| blood-periventricular__glasgow-coma-score | 4_7 | -0.47866 | -0.7217 | -0.13056 | 7.64E-05 | 1 |
| blood-periventricular__glasgow-coma-score | 8_14 | -0.48438 | -0.72937 | -0.12929 | 9.94E-05 | 1 |
| blood-periventricular__inr | 8_14 | 0.446786 | 0.074552 | 0.709742 | 0.001979 | 0 |
| blood-periventricular__pt | 8_14 | 0.421485 | 0.043653 | 0.693835 | 0.008674 | 0 |
| blood-periventricular__temp-degc | 4_7 | 0.418505 | 0.054364 | 0.684398 | 0.004649 | 0 |
| **varXY** | **win** | **r** | **lower.adj** | **upper.adj** | **p.adj** | **epi.assoc** |
| blood-periventricular__temp-degc | 8_14 | 0.382237 | 0.006572 | 0.663356 | 0.045407 | 0 |
| blood-periventricular__totalblood | 1_3 | 0.748572 | 0.528119 | 0.874493 | 6.09E-20 | 0 |
| blood-periventricular__totalblood | 4_7 | 0.751257 | 0.526609 | 0.87783 | 2.83E-19 | 0 |
| blood-periventricular__totalblood | 8_14 | 0.695292 | 0.430666 | 0.849831 | 2.1E-14 | 0 |
| bp-diastolic__bp-systolic | 1_3 | 0.515371 | 0.171261 | 0.747412 | 8.33E-06 | 0 |
| bp-diastolic__bp-systolic | 4_7 | 0.716613 | 0.487097 | 0.85354 | 1.03E-18 | 0 |
| bp-diastolic__bp-systolic | 8_14 | 0.755435 | 0.550031 | 0.874657 | 2.03E-22 | 0 |
| bp-diastolic__neutrophil | 8_14 | 0.455526 | 0.12184 | 0.696698 | 8.21E-05 | 0 |
| bp-diastolic__nlr | 8_14 | 0.385522 | 0.037829 | 0.64995 | 0.009222 | 0 |
| bp-diastolic__sii | 8_14 | 0.435731 | 0.097525 | 0.683718 | 0.000349 | 1 |
| bp-diastolic__wbc | 8_14 | 0.423013 | 0.082161 | 0.675269 | 0.000844 | 0 |
| bp-systolic__glucose | 4_7 | 0.383658 | 0.036709 | 0.648067 | 0.00959 | 0 |
| bp-systolic__glucose | 8_14 | 0.370677 | 0.022123 | 0.638885 | 0.02039 | 0 |
| bp-systolic__neutrophil | 8_14 | 0.487727 | 0.16227 | 0.717479 | 6.35E-06 | 0 |
| bp-systolic__nlr | 8_14 | 0.387425 | 0.040049 | 0.651247 | 0.008235 | 0 |
| bp-systolic__sii | 8_14 | 0.461058 | 0.128669 | 0.700317 | 5.39E-05 | 1 |
| bp-systolic__temp-degc | 4_7 | 0.413511 | 0.071918 | 0.668284 | 0.001452 | 0 |
| bp-systolic__temp-degc | 8_14 | 0.405404 | 0.063976 | 0.661869 | 0.002241 | 0 |
| bp-systolic__wbc | 8_14 | 0.458607 | 0.125637 | 0.698716 | 6.5E-05 | 0 |
| bun__bun-creat-ratio | 1_3 | 0.750555 | 0.526618 | 0.877086 | 2.15E-19 | 0 |
| bun__bun-creat-ratio | 4_7 | 0.773676 | 0.580882 | 0.884277 | 1.59E-24 | 0 |
| bun__bun-creat-ratio | 8_14 | 0.779347 | 0.588377 | 0.887966 | 9.49E-25 | 0 |
| bun__creatinine | 1_3 | 0.530357 | 0.197932 | 0.753371 | 1.25E-06 | 0 |
| bun__creatinine | 4_7 | 0.5303 | 0.221556 | 0.742413 | 8.37E-08 | 0 |
| bun__creatinine | 8_14 | 0.616953 | 0.337921 | 0.796292 | 6.94E-12 | 0 |
| bun__e-creat-clearance | 4_7 | -0.36199 | -0.6314 | -0.0146 | 0.029082 | 0 |
| bun__e-gfr | 1_3 | -0.59162 | -0.78895 | -0.28366 | 2.81E-09 | 0 |
| bun__e-gfr | 4_7 | -0.57775 | -0.77166 | -0.28556 | 5.35E-10 | 0 |
| bun__e-gfr | 8_14 | -0.58722 | -0.77913 | -0.29467 | 3.38E-10 | 0 |
| bun-creat-ratio__glucose | 4_7 | 0.378927 | 0.03404 | 0.643168 | 0.010816 | 0 |
| chloride__sodium | 1_3 | 0.710544 | 0.461814 | 0.855669 | 3.31E-16 | 1 |
| chloride__sodium | 4_7 | 0.764871 | 0.566418 | 0.87948 | 1.41E-23 | 1 |
| chloride__sodium | 8_14 | 0.703668 | 0.466905 | 0.846296 | 1.15E-17 | 1 |
| co2-content__chloride | 8_14 | -0.49107 | -0.71892 | -0.16793 | 4.16E-06 | 0 |
| creatinine__e-creat-clearance | 1_3 | -0.47003 | -0.7163 | -0.11963 | 0.000143 | 0 |
| creatinine__e-creat-clearance | 4_7 | -0.45007 | -0.69099 | -0.11916 | 8.72E-05 | 0 |
| creatinine__e-creat-clearance | 8_14 | -0.41544 | -0.67021 | -0.0731 | 0.001403 | 0 |
| creatinine__e-gfr | 1_3 | -0.72732 | -0.86514 | -0.48737 | 2.62E-17 | 0 |
| creatinine__e-gfr | 4_7 | -0.80238 | -0.89976 | -0.62888 | 6.1E-28 | 0 |
| creatinine__e-gfr | 8_14 | -0.8126 | -0.90599 | -0.64378 | 1.38E-28 | 0 |
| csf-lymph__csf-neutr | 1_3 | 0.65068 | 0.179273 | 0.879084 | 0.000283 | 1 |
| csf-lymph__csf-neutr | 4_7 | 0.755607 | 0.416308 | 0.910181 | 2.04E-09 | 1 |
| csf-lymph__csf-neutr | 8_14 | 0.746507 | 0.413462 | 0.903372 | 1.01E-09 | 1 |
| csf-lymph__csf-rbc | 1_3 | 0.576281 | 0.061715 | 0.848832 | 0.012328 | 1 |
| csf-lymph__csf-wbc | 1_3 | 0.764976 | 0.390575 | 0.922228 | 6.54E-08 | 1 |
| **varXY** | **win** | **r** | **lower.adj** | **upper.adj** | **p.adj** | **epi.assoc** |
| csf-lymph__csf-wbc | 4_7 | 0.829845 | 0.568144 | 0.939115 | 1.25E-13 | 1 |
| csf-lymph__csf-wbc | 8_14 | 0.840772 | 0.603332 | 0.941288 | 1.69E-15 | 1 |
| csf-neutr__csf-rbc | 1_3 | 0.743842 | 0.348293 | 0.914533 | 4.27E-07 | 1 |
| csf-neutr__csf-wbc | 1_3 | 0.977519 | 0.927831 | 0.993119 | 2.97E-32 | 1 |
| csf-neutr__csf-wbc | 4_7 | 0.981503 | 0.946176 | 0.993718 | 1.44E-41 | 1 |
| csf-neutr__csf-wbc | 8_14 | 0.962505 | 0.896349 | 0.986732 | 9.27E-35 | 1 |
| csf-rbc__csf-wbc | 1_3 | 0.746403 | 0.353326 | 0.915474 | 3.44E-07 | 1 |
| e-creat-clearance__e-gfr | 1_3 | 0.709152 | 0.459615 | 0.854913 | 4.17E-16 | 0 |
| e-creat-clearance__e-gfr | 4_7 | 0.70366 | 0.469152 | 0.845468 | 5.77E-18 | 0 |
| e-creat-clearance__e-gfr | 8_14 | 0.655291 | 0.392429 | 0.819195 | 4.4E-14 | 0 |
| eosinophil__nlr | 4_7 | -0.40688 | -0.66225 | -0.06682 | 0.001861 | 1 |
| eye-opening__glasgow-coma-score | 1_3 | 0.867719 | 0.732087 | 0.937177 | 1.71E-33 | 1 |
| eye-opening__glasgow-coma-score | 4_7 | 0.850405 | 0.712325 | 0.925108 | 3.89E-35 | 1 |
| eye-opening__glasgow-coma-score | 8_14 | 0.839395 | 0.691337 | 0.919801 | 9.55E-33 | 1 |
| eye-opening__glucose | 4_7 | -0.38729 | -0.64891 | -0.04376 | 0.006504 | 1 |
| eye-opening__temp-degc | 8_14 | -0.36568 | -0.63543 | -0.01639 | 0.02709 | 1 |
| glasgow-coma-score__calcium | 8_14 | 0.392314 | 0.047179 | 0.653767 | 0.00561 | 1 |
| glasgow-coma-score__chloride | 8_14 | -0.39323 | -0.6544 | -0.04823 | 0.005306 | 1 |
| glasgow-coma-score__glucose | 4_7 | -0.37813 | -0.64261 | -0.03313 | 0.011342 | 1 |
| glasgow-coma-score__temp-degc | 4_7 | -0.44415 | -0.68859 | -0.10908 | 0.000171 | 1 |
| glasgow-coma-score__temp-degc | 8_14 | -0.53806 | -0.74853 | -0.22909 | 5.5E-08 | 1 |
| glucose__csf-glucose | 1_3 | 0.605087 | 0.105555 | 0.860772 | 0.003209 | 0 |
| glucose__csf-glucose | 4_7 | 0.701086 | 0.315716 | 0.887915 | 3.56E-07 | 0 |
| glucose__csf-glucose | 8_14 | 0.573216 | 0.126747 | 0.826556 | 0.000777 | 0 |
| hct__calcium | 4_7 | 0.50708 | 0.191164 | 0.727802 | 7.55E-07 | 0 |
| hct__calcium | 8_14 | 0.405179 | 0.060862 | 0.663322 | 0.002739 | 0 |
| hct__hgb | 1_3 | 0.956177 | 0.906699 | 0.979696 | 5.77E-60 | 0 |
| hct__hgb | 4_7 | 0.971657 | 0.941975 | 0.986263 | 1.05E-80 | 0 |
| hct__hgb | 8_14 | 0.97363 | 0.945499 | 0.987335 | 8.42E-81 | 0 |
| hct__rbc | 1_3 | 0.694914 | 0.43585 | 0.847637 | 5.83E-15 | 0 |
| hct__rbc | 4_7 | 0.800234 | 0.625247 | 0.898611 | 1.15E-27 | 0 |
| hct__rbc | 8_14 | 0.836377 | 0.685251 | 0.918452 | 5.24E-32 | 0 |
| hct__rdw | 8_14 | -0.5297 | -0.74396 | -0.21669 | 1.48E-07 | 0 |
| hgb__calcium | 4_7 | 0.49179 | 0.171498 | 0.718059 | 2.94E-06 | 0 |
| hgb__calcium | 8_14 | 0.397131 | 0.05134 | 0.657894 | 0.004548 | 0 |
| hgb__mchc | 1_3 | 0.418314 | 0.055821 | 0.683372 | 0.00427 | 0 |
| hgb__mchc | 8_14 | 0.477216 | 0.148951 | 0.710741 | 1.51E-05 | 0 |
| hgb__rbc | 1_3 | 0.671982 | 0.400356 | 0.835059 | 1.77E-13 | 0 |
| hgb__rbc | 4_7 | 0.794011 | 0.614746 | 0.895271 | 6.88E-27 | 0 |
| hgb__rbc | 8_14 | 0.804125 | 0.629239 | 0.901505 | 1.75E-27 | 0 |
| hgb__rdw | 1_3 | -0.41472 | -0.68102 | -0.05154 | 0.005285 | 0 |
| hgb__rdw | 4_7 | -0.35751 | -0.62823 | -0.00957 | 0.037394 | 0 |
| hgb__rdw | 8_14 | -0.58635 | -0.7786 | -0.29347 | 3.73E-10 | 0 |
| inr__calcium | 4_7 | -0.46604 | -0.70215 | -0.13759 | 2.85E-05 | 0 |
| inr__pt | 1_3 | 0.94399 | 0.881967 | 0.973874 | 1.8E-54 | 0 |
| **varXY** | **win** | **r** | **lower.adj** | **upper.adj** | **p.adj** | **epi.assoc** |
| inr__pt | 4_7 | 0.963781 | 0.925959 | 0.982459 | 2.88E-73 | 0 |
| inr__pt | 8_14 | 0.949814 | 0.896134 | 0.976101 | 1.04E-60 | 0 |
| lymphocyte__monocyte | 1_3 | 0.381637 | 0.01237 | 0.659303 | 0.034387 | 0 |
| lymphocyte__mpvr | 8_14 | -0.38188 | -0.64745 | -0.03362 | 0.011457 | 0 |
| lymphocyte__nlr | 1_3 | -0.75611 | -0.8804 | -0.53462 | 1.07E-19 | 0 |
| lymphocyte__nlr | 4_7 | -0.66816 | -0.82519 | -0.41525 | 2.52E-15 | 0 |
| lymphocyte__nlr | 8_14 | -0.65507 | -0.81906 | -0.39211 | 4.54E-14 | 0 |
| lymphocyte__platelet | 4_7 | 0.358274 | 0.010436 | 0.628766 | 0.035842 | 0 |
| lymphocyte__plr | 1_3 | -0.69565 | -0.84804 | -0.43699 | 5.21E-15 | 0 |
| lymphocyte__plr | 4_7 | -0.74038 | -0.86601 | -0.52683 | 3.84E-21 | 0 |
| lymphocyte__plr | 8_14 | -0.57541 | -0.772 | -0.27834 | 1.3E-09 | 0 |
| lymphocyte__sii | 1_3 | -0.54112 | -0.75984 | -0.21235 | 4.83E-07 | 1 |
| lymphocyte__sii | 4_7 | -0.45943 | -0.69713 | -0.13073 | 4.24E-05 | 1 |
| mch__mchc | 1_3 | 0.414256 | 0.052687 | 0.679798 | 0.004931 | 0 |
| mch__mchc | 4_7 | 0.356477 | 0.008427 | 0.627487 | 0.039575 | 0 |
| mch__mchc | 8_14 | 0.510888 | 0.192069 | 0.732164 | 8.48E-07 | 0 |
| mch__mcv | 1_3 | 0.901059 | 0.796483 | 0.953296 | 1.1E-40 | 0 |
| mch__mcv | 4_7 | 0.888162 | 0.780785 | 0.94458 | 7.46E-43 | 0 |
| mch__mcv | 8_14 | 0.861355 | 0.729883 | 0.931375 | 3.01E-36 | 0 |
| mch__platelet | 4_7 | -0.36927 | -0.63648 | -0.02291 | 0.019148 | 0 |
| mch__rbc | 1_3 | -0.39878 | -0.67061 | -0.03254 | 0.013357 | 0 |
| mch__rdw | 1_3 | -0.49686 | -0.73218 | -0.15559 | 1.68E-05 | 0 |
| mch__rdw | 4_7 | -0.51156 | -0.73064 | -0.19697 | 5E-07 | 0 |
| mch__rdw | 8_14 | -0.5328 | -0.7459 | -0.22075 | 1.1E-07 | 0 |
| mchc__rdw | 1_3 | -0.39546 | -0.66748 | -0.03031 | 0.014737 | 0 |
| mchc__rdw | 8_14 | -0.52804 | -0.74291 | -0.21452 | 1.73E-07 | 0 |
| mcv__rbc | 1_3 | -0.5038 | -0.73724 | -0.16291 | 1.13E-05 | 0 |
| mcv__rbc | 4_7 | -0.36822 | -0.63575 | -0.02171 | 0.020347 | 0 |
| mcv__rdw | 1_3 | -0.40245 | -0.67208 | -0.03859 | 0.009888 | 0 |
| mcv__rdw | 4_7 | -0.40004 | -0.65762 | -0.05873 | 0.002908 | 0 |
| monocyte__anion-gap | 4_7 | 0.363245 | 0.016033 | 0.632279 | 0.027088 | 0 |
| monocyte__neutrophil | 4_7 | 0.625848 | 0.353262 | 0.800484 | 1.29E-12 | 0 |
| monocyte__neutrophil | 8_14 | 0.623844 | 0.346524 | 0.80088 | 3.6E-12 | 0 |
| monocyte__nlr | 8_14 | 0.416875 | 0.074806 | 0.671165 | 0.001276 | 0 |
| monocyte__plr | 1_3 | -0.38095 | -0.65884 | -0.01158 | 0.035652 | 0 |
| monocyte__sii | 4_7 | 0.356528 | 0.00846 | 0.627538 | 0.039488 | 1 |
| monocyte__sii | 8_14 | 0.381398 | 0.033066 | 0.64711 | 0.011785 | 1 |
| monocyte__wbc | 1_3 | 0.415845 | 0.052882 | 0.681755 | 0.004943 | 0 |
| monocyte__wbc | 4_7 | 0.697258 | 0.45932 | 0.841835 | 1.84E-17 | 0 |
| monocyte__wbc | 8_14 | 0.626782 | 0.35075 | 0.802607 | 2.44E-12 | 0 |
| mpv__e-gfr | 4_7 | -0.37619 | -0.64127 | -0.03089 | 0.012734 | 0 |
| mpv__mpvr | 1_3 | 0.714232 | 0.467675 | 0.857666 | 1.77E-16 | 0 |
| mpv__mpvr | 4_7 | 0.567644 | 0.27172 | 0.765498 | 1.68E-09 | 0 |
| mpv__mpvr | 8_14 | 0.492809 | 0.168739 | 0.720729 | 4.14E-06 | 0 |
| mpv__platelet | 1_3 | -0.54046 | -0.75872 | -0.2131 | 4.3E-07 | 0 |
| **varXY** | **win** | **r** | **lower.adj** | **upper.adj** | **p.adj** | **epi.assoc** |
| mpvr__bun | 4_7 | 0.36749 | 0.020879 | 0.635242 | 0.021215 | 0 |
| mpvr__bun | 8_14 | 0.391852 | 0.04522 | 0.654266 | 0.00629 | 0 |
| mpvr__e-gfr | 4_7 | -0.39062 | -0.65119 | -0.04765 | 0.005287 | 0 |
| mpvr__e-gfr | 8_14 | -0.3993 | -0.66019 | -0.05243 | 0.00435 | 0 |
| mpvr__platelet | 1_3 | -0.96609 | -0.98428 | -0.92762 | 7.79E-67 | 0 |
| mpvr__platelet | 4_7 | -0.96073 | -0.98091 | -0.92006 | 1.39E-71 | 0 |
| mpvr__platelet | 8_14 | -0.96016 | -0.9808 | -0.91827 | 1.61E-69 | 0 |
| neutrophil__glucose | 4_7 | 0.369954 | 0.023678 | 0.63697 | 0.018408 | 0 |
| neutrophil__nlr | 1_3 | 0.583565 | 0.270759 | 0.784905 | 8.12E-09 | 0 |
| neutrophil__nlr | 4_7 | 0.701622 | 0.466016 | 0.844313 | 8.38E-18 | 0 |
| neutrophil__nlr | 8_14 | 0.630493 | 0.356109 | 0.804784 | 1.48E-12 | 0 |
| neutrophil__sii | 1_3 | 0.588232 | 0.277319 | 0.787623 | 4.99E-09 | 1 |
| neutrophil__sii | 4_7 | 0.784343 | 0.59857 | 0.890057 | 9.85E-26 | 1 |
| neutrophil__sii | 8_14 | 0.736366 | 0.517245 | 0.864885 | 2.97E-20 | 1 |
| neutrophil__wbc | 1_3 | 0.966147 | 0.927505 | 0.984359 | 2.74E-66 | 0 |
| neutrophil__wbc | 4_7 | 0.941597 | 0.882321 | 0.971468 | 1.45E-60 | 0 |
| neutrophil__wbc | 8_14 | 0.949144 | 0.896276 | 0.975415 | 6.97E-63 | 0 |
| nlr__glucose | 4_7 | 0.430576 | 0.095294 | 0.678119 | 0.000366 | 0 |
| nlr__plr | 1_3 | 0.668481 | 0.395017 | 0.833121 | 2.91E-13 | 0 |
| nlr__plr | 4_7 | 0.66507 | 0.41065 | 0.823407 | 4.11E-15 | 0 |
| nlr__plr | 8_14 | 0.597037 | 0.308405 | 0.785004 | 1.06E-10 | 0 |
| nlr__sii | 1_3 | 0.832651 | 0.667478 | 0.919727 | 4.45E-28 | 1 |
| nlr__sii | 4_7 | 0.881106 | 0.767792 | 0.940971 | 3.23E-41 | 1 |
| nlr__sii | 8_14 | 0.7912 | 0.607301 | 0.894626 | 6.7E-26 | 1 |
| nlr__wbc | 1_3 | 0.43645 | 0.077857 | 0.695049 | 0.001384 | 0 |
| nlr__wbc | 4_7 | 0.48431 | 0.161977 | 0.713257 | 5.59E-06 | 0 |
| nlr__wbc | 8_14 | 0.424094 | 0.083457 | 0.675992 | 0.000785 | 0 |
| platelet__bun | 8_14 | -0.36807 | -0.63793 | -0.01769 | 0.025546 | 0 |
| platelet__e-gfr | 8_14 | 0.370848 | 0.019408 | 0.640725 | 0.023561 | 0 |
| plr__mpvr | 1_3 | -0.46231 | -0.71145 | -0.10993 | 0.000246 | 0 |
| plr__mpvr | 8_14 | -0.44812 | -0.69187 | -0.11269 | 0.000143 | 0 |
| plr__platelet | 1_3 | 0.436117 | 0.077463 | 0.69483 | 0.001413 | 0 |
| plr__platelet | 8_14 | 0.48411 | 0.157681 | 0.715162 | 8.58E-06 | 0 |
| plr__sii | 1_3 | 0.844617 | 0.689246 | 0.925722 | 8.97E-30 | 1 |
| plr__sii | 4_7 | 0.759251 | 0.557252 | 0.876404 | 5.42E-23 | 1 |
| plr__sii | 8_14 | 0.787629 | 0.601294 | 0.892715 | 1.75E-25 | 1 |
| pt__calcium | 4_7 | -0.45284 | -0.69354 | -0.12119 | 7.94E-05 | 0 |
| rbc__calcium | 4_7 | 0.424961 | 0.088505 | 0.674376 | 0.000544 | 0 |
| rbc__calcium | 8_14 | 0.397311 | 0.05154 | 0.658023 | 0.0045 | 0 |
| sii__glucose | 4_7 | 0.381651 | 0.037208 | 0.645039 | 0.009186 | 1 |
| sii__mpvr | 1_3 | -0.4217 | -0.68556 | -0.0599 | 0.003478 | 1 |
| sii__mpvr | 8_14 | -0.4387 | -0.68567 | -0.10114 | 0.000283 | 1 |
| sii__platelet | 1_3 | 0.417467 | 0.054812 | 0.682818 | 0.004492 | 1 |
| sii__platelet | 4_7 | 0.354199 | 0.005846 | 0.625888 | 0.044884 | 1 |
| sii__platelet | 8_14 | 0.522838 | 0.207675 | 0.739663 | 2.82E-07 | 1 |
| **varXY** | **win** | **r** | **lower.adj** | **upper.adj** | **p.adj** | **epi.assoc** |
| sii__wbc | 1_3 | 0.464645 | 0.11286 | 0.712918 | 0.000209 | 1 |
| sii__wbc | 4_7 | 0.630439 | 0.359871 | 0.803196 | 6.86E-13 | 1 |
| sii__wbc | 8_14 | 0.600004 | 0.312571 | 0.786777 | 7.39E-11 | 1 |
| totalblood__best-motor-response | 8_14 | -0.42729 | -0.69364 | -0.05816 | 0.004122 | 0 |
| totalblood__best-verbal-response | 1_3 | -0.44958 | -0.70341 | -0.09405 | 0.000586 | 1 |
| totalblood__best-verbal-response | 4_7 | -0.60192 | -0.79553 | -0.29678 | 1.14E-09 | 1 |
| totalblood__best-verbal-response | 8_14 | -0.56082 | -0.77513 | -0.23078 | 2.04E-07 | 1 |
| totalblood__csf-rbc | 8_14 | 0.535756 | 0.073256 | 0.808603 | 0.00558 | 1 |
| totalblood__glasgow-coma-score | 1_3 | -0.43709 | -0.69546 | -0.07863 | 0.001329 | 1 |
| totalblood__glasgow-coma-score | 4_7 | -0.48109 | -0.72322 | -0.13364 | 6.39E-05 | 1 |
| totalblood__glasgow-coma-score | 8_14 | -0.50437 | -0.74155 | -0.15513 | 2.28E-05 | 1 |
| totalblood__inr | 8_14 | 0.393866 | 0.010752 | 0.676148 | 0.03817 | 0 |
| wbc__platelet | 4_7 | 0.399743 | 0.058383 | 0.657408 | 0.002963 | 0 |
| wbc__platelet | 8_14 | 0.396453 | 0.050551 | 0.65743 | 0.004742 | 0 |

For each combination of variables (“varXY”), mean *r* values with confidence interval upper (“upper.adj”) and lower (“lower.adj”) bounds as well as *P* values (“p.adj”) adjusted for multiple comparisons are shown together with their corresponding time windows (“win”), shown as “start-day_end-day”. Only those correlations with adjusted *P*<0.05 are shown in this table. The “epi.assoc” column indicates whether either of the variables in the pair were associated with post-SAH epilepsy. Composite Glasgow Coma Scores and component sub-scores (“best-motor-response”, “best-verbal-response”, “eye-opening”) were analyzed as separate variables. Cell counts are indicated with the cell name/type (e.g. “platelet”, “monocyte”, “neutrophil”); electrolyte levels (e.g. “sodium”) and other routine serum labs (e.g. “glucose”) are similarly indicated. “inr” = international normalized ratio, “pt” = prothrombin time. “mpvr” = mean platelet volume ratio. “bp” = blood pressure.

**SUPPLEMENTARY METHODS**

**CT Image Analysis**

We used the external deep learning (DL) model developed by Thanellas et al. [1] to measure blood volumes that avoids potential false positives which frequently develop following the placement of EVDs or aneurysm clips.

**Construction of Custom Label Atlases**

Preliminary Proof-of-Concept Atlas

As a proof of concept, we initially constructed a label atlas in which naïvely divides the brain into 16 subregions. We then extended this approach to generate the label atlas described in the article. Here, we provide foundational detail on how we constructed a proof-of-concept atlas, followed by detail on the construction of the anatomical atlas used in our study.

The CT Template was first divided along the inferior-superior axis into 4 (roughly) equal “axial slabs,” each of which is split into four (roughly) equal quadrants using longitudinal fissure to define the left and right sides and the point of maximum width along the left-right axis to separate anterior and posterior areas.

To procedure to create the label atlas is as follows:

1. Created a binary mask of the CT Template
2. Used fixed x-y coordinates and slice levels to isolate subregions of the binary Template mask automatically.

The inferior and superior limits were defined to exclude sections within ≈10 mm of the skull base and vertex, respectively. The purpose was to limit sampling of areas which are highly sensitive to beam hardening artifact due to the presence of skull.

When combined with co-registered segmentations for the (lateral) ventricles, the label atlas can be updated (i.e. adjusted) to account for true position and size of the lateral ventricles on each individual image. The procedure is as follows:

1. Multiply the pixels in the ventricle mask by -1 (i.e. change the value of all pixels defining the ventricles from 1 to -1)
2. Set all 0 pixels in the ventricle mask to 1
3. Multiply the label mask by the ventricle mask
4. Assign a unique integer label to all negative-valued pixels in this mask.

**
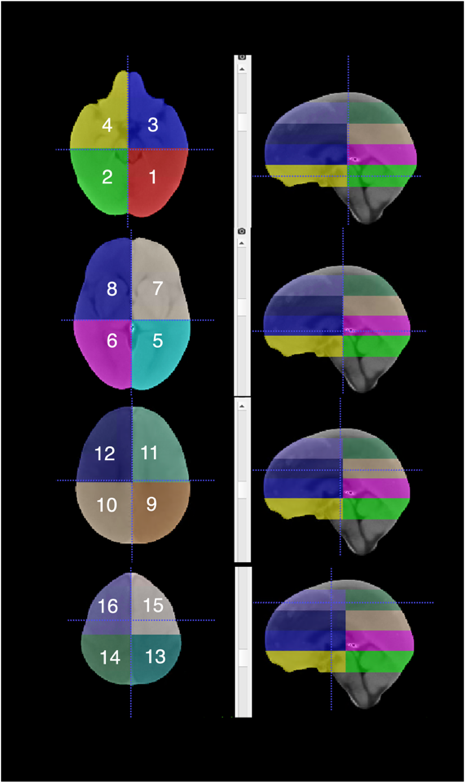
**

Image above shows cross-sectional images preliminary custom label atlas dividing the brain into 16 subregions based on midlines drawn in the sagittal and axial planes. The 16 subregions labeled by color overlaid on the CT Template in both axial (left side) and sagittal (right side) sections. The numerical values for each label are shown for reference. The position of the crosshairs (blue dotted lines) shown on the sagittal images indicate the corresponding level of each axial image. Labels were designed so that odd numbers designate left-sided subregions and even numbers designate right-sided ones.

Construction of Anatomical Compartment Atlas

For this study, we constructed a new label atlas to subdivide the (Template) brain into four anatomical compartments relevant to SAH patients: cisternal spaces, pericortical regions capturing sulcal spaces and grey matter areas, parenchymal areas covering white matter and deeper brain structures, and periventricular regions encompassing the space within and proximal to the ventricles. We defined these compartments semi-automatically using the correction mask described above which we developed as part of our automated lateral ventricle segmentation algorithm [2] as well as MRI-derived segmentations of the grey matter, white matter, and CSF spaces which distributed with template CT developed by Rorden et al. [3]. Figure 2 (main text) shows four example images of the CT Template with colored overlays indicating cisternal (red), pericortical (green), parenchymal (blue), and periventricular (yellow) regions. We ultimately quantified and further analyzed six parameters from each patient CT and used them in: total blood volume, total ventricular volume, as well as regional blood volume in the cisternal, pericortical, parenchymal, and periventricular areas.

**FPCA, Data Imputation, and Logistic Regression**

FPCA is a well-described method for extracting concise sets of features from time series data. We implemented FPCA using the ‘fdapace’ package in R [4]. Since the algorithm did not reliably converge on the 2-day binned data and is capable of handling sparse data well, we used 1-day binned data as was used for the SSANOVA. The time series curve for each variable within each patient was first reduced to a set of coefficients (“FPC scores”) corresponding to parameters of random functions which capture the information contained in the original curve. FPC scores were then assigned numerical labels in decreasing order based on the proportion of variance. We used the first 2 FPC scores for each variable, which typically explained ≥95% of the variance.

Multivariate imputation by chained equations (MICE) was implemented via the ‘mice’ package [5] in R to generate completed datasets of FPC scores and/or clinical covariate measures for all variables. Given that CSF data was not routinely collected at daily intervals and thus unavailable for a large portion (>47%) of possible time points, MICE was implemented using 60 imputations with 20 iterations per imputation. The distributions of imputed variables were compared with those of the non-imputed data to confirm the suitability of imputed values for further analysis.

Longitudinal variables were grouped by similarity, combined with significant (*P*<0.05) clinical covariates, and assessed via Pearson correlation to ensure that highly collinear variables were never included in the same model. Multicollinearity was assessed for each model by computing the variance inflation factor (VIF), and all models with VIF≥5 for at least one variable excluded.

**Correlation Analysis**

Only variables present in at least 10% of patients with no more than 62% missing data were included. *P*-values adjusted for multiple comparisons testing were computed for all correlation coefficients and results were visualized with heatmaps using the ’corrplot’ package [6] in R [7].

**REFERENCES**

[1] Thanellas A, Peura H, Lavinto M, Ruokola T, Vieli M, Staartjes VE, et al. Development and External Validation of a Deep Learning Algorithm to Identify and Localize Subarachnoid Hemorrhage on CT Scans. Neurology 2023. https://doi.org/10.1212/WNL.0000000000201710.

[2] Butler M, Shah P, Ozgen B, Michals EA, Geraghty JR, Testai FD, et al. Automated segmentation of ventricular volumes and subarachnoid hemorrhage from computed tomography images: Evaluation of a rule-based pipeline approach. Neuroradiol J 2024:19714009241260791. https://doi.org/10.1177/19714009241260791.

[3] Rorden C, Bonilha L, Fridriksson J, Bender B, Karnath H-O. Age-specific CT and MRI templates for spatial normalization. NeuroImage 2012;61:957–65. https://doi.org/10.1016/j.neuroimage.2012.03.020.

[4] Zhou Y, Bhattacharjee S, Carroll C, Chen Y, Dai X, Fan J, et al. fdapace: Functional Data Analysis and Empirical Dynamics. 2022.

[5] Buuren S van, Groothuis-Oudshoorn K. mice: Multivariate Imputation by Chained Equations in R. Journal of Statistical Software 2011;45:1–67. https://doi.org/10.18637/jss.v045.i03.

[6] Wei T, Simko V. R package “corrplot”: Visualization of a Correlation Matrix. 2021.

[7] R Core Team. R: A Language and Environment for Statistical Computing. Vienna, Austria: R Foundation for Statistical Computing; 2023.

**OTHER SUPPLEMENTARY MATERIAL**

**Date: __/__/____**

**Survey: Assessment of Epilepsy-Related Outcomes Following Subarachnoid Hemorrhage**

***INSTRUCTIONS: As you complete the questions below, please follow the instructions given in the grey boxes.*** ***You may not have to complete all of the survey questions.***

***If you have any questions about completing this survey or the overall study, please feel free to call the UIC Neurology Research Team at 312-413-1882.***

1. **Are you currently taking any medications to treat your seizures (antiepileptic medications)?**
   - **Yes**
   - **No**
   - **Refused**
   - **Don’t know**

*If your answer to Question 1 is* ***YES****:*

*Continue with the questions below. _________________________________________________________*

*If your answer to Question 1 is* ***NO*** *or* ***DON’T KNOW****:*

*Skip to Question 4 and continue from that question.*

1. **How many medications are you currently taking to treat your seizures (antiepileptic medications)?**
   - **1**
   - **2**
   - **3**
   - **4 or more medications**
   - **Don’t know/ Unknown**
2. **What are the name(s) of the medicine(s) you currently take to treat your seizures (antiepileptic medications)? Include as many as apply.**
   - **Don’t know/ Unknown**

| **Generic name** | **Brand name** | **Brand name** |
| --- | --- | --- |
| **☐ Carbamazepine** | **☐ Tegretol** |  |
| **☐ Carbamazepine-XR** | **☐ Tegretol XR** | **☐ Carbatrol** |
| **☐ Clobazam** | **☐ Onfi** |  |
| **☐ Clonazepam** | **☐ Klonopin** |  |
| **☐ Diazepam** | **☐ Valium** | **☐ Diastat** |
| **☐ Divalproex Sodium** | **☐ Depakote** | **☐ Depacon** |
| **☐ Divalproex Sodium-ER** | **☐ Depakote ER** |  |
| **☐ Eslicarbazepine Acetate** | **☐ Aptiom** |  |
| **☐ Ethosuximide** | **☐ Zarontin** |  |
| **☐ Felbamate** | **☐ Felbatol** |  |
| **☐ Gabapentin** | **☐ Neurontin** |  |
| **☐ Lacosamide** | **☐ Vimpat** |  |
| **☐ Lamotrigine** | **☐ Lamictal** |  |
| **☐ Lamotrigine ER** | **☐ Lamictal XR** |  |
| **☐ Levetiracetam** | **☐ Keppra** |  |
| **☐ Levetiracetam XR** | **☐ Keppra XR** |  |
| **☐ Lorazepam** | **☐ Ativan** |  |
| **☐ Midazolam** | | |
| **☐ Oxcarbazepine** | **☐ Trileptal** |  |
| **☐ Oxcarbazepine XR** | **☐ Oxtellar XR** |  |
| **☐ Perampanel** | **☐ Fycompa** |  |
| **☐ Phenobarbital** | | |
| **☐ Phenytoin** | **☐ Dilantin** | **☐ Phenytek** |
| **☐ Pregabalin** | **☐ Lyrica** |  |
| **☐ Primidone** | **☐ Mysoline** |  |
| **☐ Rufinamide** | **☐ Banzel** |  |
| **☐ Tiagabine Hydrochloride** | **☐ Gabitrl** |  |
| **☐ Topiramate- Topamax** | **☐ Topamax** |  |
| **☐ Topiramate Extended Release Capsules** | **☐ Qudexy XR** |  |
| **☐ Topiramate XR** | **☐ Trokendi XR** |  |
| **☐ Valproic Acid** | **☐ Depakene** |  |
| **☐ Vigabatrin** | **☐ Sabril** |  |
| **☐ Zonisamide** | **☐ Zonegran** |  |

1. **Since your subarachnoid hemorrhage, please fill in the corresponding box if you have ever had, or if anyone has ever told you that you had, any of the following…**
   - **A seizure, convulsion, fit or spell under any circumstances?**
   - **Uncontrolled movements of part or all of your body such as twitching, jerking, shaking or going limp?**
   - **An unexplained change in your mental state or level of awareness; or an episode of “spacing out” that you could not control?**
   - **Have you ever lost consciousness?**
   - **Have you ever had attacks in which you fall with loss of consciousness?**
   - **Have you ever had attacks in which you fall and become pale?**
   - **Have you ever had attacks in which you fall and bite your tongue?**
   - **Have you ever had attacks in which you fall and lose control of your bladder?**
   - **Have you ever had attacks in which you lose contact with the surroundings and experience abnormal smells?**
   - **Have you ever noticed any unusual body movements or feelings when exposed to strobe lights, video games, flickering lights, or sun glare?**
   - **Shortly after waking up, either in the morning or after a nap, have you ever noticed uncontrollable jerking or clumsiness, such as dropping things or things suddenly “flying” from your hands?**
   - **Have you ever had any other type of repeated unusual spells?**
2. **In the past year have you seen a neurologist or epilepsy specialist for a seizure disorder/epilepsy?**
   - **Yes**
   - **No**
   - **Refused**
   - **Don’t know**
3. **Since your Subarachnoid Hemorrhage, have you ever been told by a doctor or other health professional that you have a seizure disorder or epilepsy?**
   - **Yes**
   - **No**
   - **Refused**
   - **Don’t know**

| ***If your answer to Question 6 is YES:***  ***Continue with the questions below.***    ***If you answer to Question 6 is NO or DON’T KNOW:***  ***Your participation in this study is complete. You do not need to fill out any of the questions below. You also do not need to complete the other attached survey, “Liverpool Seizure Severity Scale 2.0”. Please send these surveys back to the UIC Neurology Research Team using the prepaid envelope included in this package. If you have any future questions, please feel free to call our office at 312-413-1882. Thank you for your time and participation!*** |
| --- |

1. **When were you diagnosed with epilepsy?**
   - **In the past year**
   - **1-2 years ago**
   - **3-4 years ago**
   - **5-6 years ago**
   - **7-8 years ago**
   - **9 or more years ago**
   - **Don’t know/ Unknown**
2. **About how many seizures of any type have you had in the past year?**
   - **None**
   - **One**
   - **Two or Three**
   - **Between Four and Ten**
   - **More than 10**
   - **Refused**
   - **Don't know/ Unknown**
3. **How long ago was your most recent seizure?**
   - **Within the past week**
   - **2 to 4 weeks ago**
   - **2 to 3 months ago**
   - **4 to 6 months ago**
   - **7 to 12 months ago**
   - **Don’t know/ Unknown**
4. **Have you ever had surgery, a vagus nerve stimulator (VNS), or any treatments other than medications for your epilepsy? Include as many as apply.**
   - **Yes- Surgery**
   - **Yes- VNS**
   - **Yes- Other; Please describe here _____________________**
   - **No**
   - **Don’t know/ Unknown**

| ***If your answer to Question 10 is YES:***  ***Continue with Question 11 below.***    ***If you answer to Question 10 is NO or DON’T KNOW/ UNKNOWN:***  ***Do NOT complete Question 11. Turn the page and complete the attached “Liverpool Seizure Severity Scale 2.0” survey. Upon completion of the attached “Liverpool Seizure Severity Scale 2.0” Survey, your participation in this study will be complete. Please send these surveys back to the UIC Neurology Research Team using the prepaid envelope included in this package. If you have any further questions, please feel free to call our office at 312-413-1882.***  ***Thank you for your time and participation!*** |
| --- |

1. **Did any of the treatment(s) that you used besides medications (i.e. as listed in Question 8- surgery, VNS, etc.) cause a long-term reduction or complete remission of your seizures? Include as many as apply.**
   - **Yes- Surgery**
   - **Yes- VNS**
   - **Yes- Other; Please describe here________________________**
   - **No**
   - **Don’t know/ Unknown**

| ***Upon your complete of Question 11, turn the page and complete the attached “Liverpool Seizure Severity Scale 2.0” Survey.***  ***Upon your complete of the attached “Liverpool Seizure Severity Scale 2.0” Survey, your participation in this study will be complete. Please send these surveys back to UIC Neurology Research Team using the prepaid envelope included in this package. If you have any future questions, please feel free to call our office at 312-413-1882.***  ***Thank you for your time and participation in this study!*** |
| --- |
